# Subset of Cortical Layer 6b Neurons Selectively Innervates Higher Order Thalamic Nuclei in Mice

**DOI:** 10.1093/cercor/bhy036

**Published:** 2018-02-22

**Authors:** Anna Hoerder-Suabedissen, Shuichi Hayashi, Louise Upton, Zachary Nolan, Diana Casas-Torremocha, Eleanor Grant, Sarada Viswanathan, Patrick O Kanold, Francisco Clasca, Yongsoo Kim, Zoltán Molnár

**Affiliations:** 1 Department of Physiology, Anatomy and Genetics, University of Oxford, Oxford OX1 3QX, UK; 2 Neural and Behavioral Sciences, Pennsylvania State University, 500 University Drive, Hershey, PA 17033, USA; 3 Department of Anatomy, Histology and Neuroscience, School of Medicine, Autónoma University, Madrid, Spain; 4 Department of Biology, University of Maryland, 1116 Biosciences Building,College Park, MD 20742, USA

## Abstract

The thalamus receives input from 3 distinct cortical layers, but input from only 2 of these has been well characterized. We therefore investigated whether the third input, derived from layer 6b, is more similar to the projections from layer 6a or layer 5. We studied the projections of a restricted population of deep layer 6 cells (“layer 6b cells”) taking advantage of the transgenic mouse Tg(Drd1a-cre)FK164Gsat/Mmucd (Drd1a-Cre), that selectively expresses Cre-recombinase in a subpopulation of layer 6b neurons across the entire cortical mantle. At P8, 18% of layer 6b neurons are labeled with Drd1a-Cre::tdTomato in somatosensory cortex (SS), and some co-express known layer 6b markers. Using Cre-dependent viral tracing, we identified topographical projections to higher order thalamic nuclei. VGluT1+ synapses formed by labeled layer 6b projections were found in posterior thalamic nucleus (Po) but not in the (pre)thalamic reticular nucleus (TRN). The lack of TRN collaterals was confirmed with single-cell tracing from SS. Transmission electron microscopy comparison of terminal varicosities from layer 5 and layer 6b axons in Po showed that L6b varicosities are markedly smaller and simpler than the majority from L5. Our results suggest that L6b projections to the thalamus are distinct from both L5 and L6a projections.

## Introduction

In rodents, layer 6 is comprised of 2 cytoarchitectonically distinct sublayers; the larger, upper layer 6a, and the lower compact layer 6b. Transcriptomic analysis revealed clear distinctions between them (Hoerder-Suabedissen et al. 2009; Belgard et al. 2011; Oeschger et al. 2012; Marx and Feldmeyer 2013; Tasic et al. 2016; Chevée et al. 2018). Layer 6b neurons in rodent and interstitial white matter neurons in primate are considered as the remnant of the largely transient subplate neurons (Kostovic and Rakic 1990; Lein et al. 1999; Kanold et al. 2003; Chen et al. 2012; Tolner et al. 2012; Marx et al. 2017). Layer 6b neurons have heterogeneous morphology (Marx and Feldmeyer 2013), developmental origin and birthdates (Hoerder-Suabedissen and Molnár 2013; Pedraza et al. 2014).

The adult layer 6b neurons are the only cortical neurons responsive to orexin, a peptide that is wake-promoting and produced in the lateral hypothalamus (Bayer et al., 2004; Wenger Combremont et al., 2016) and layer 6b cells also selectively respond to the neuromodulators neurotensin (Case et al. 2016) and cholecystokinin (CCK) (Chung et al. 2009). Layer 6b cells produce slow oscillations that could contribute to slow-wave sleep (Crunelli et al. 2015). Layer 6b has been proposed to function as an orexin-gated feed-forward loop potentiating thalamocortical arousal (Hay et al. 2015), based on its intracortical connections. However, there are also significant corticofugal projections from layer 6b, which may be of considerable importance.

The corticofugal projections from layers 5 and 6 have distinct relationships to first order (also called relay) nuclei and higher order (also called association) nuclei of the thalamus (Sherman and Guillery 1996; Jones 2002). Projections from cortical layer 6a provide abundant inputs to all thalamic nuclei (Reichova and Sherman 2004) and modulate how sensory information is relayed to the cortex (Olsen et al. 2012; Lam and Sherman 2013; Crandall et al. 2015). Layer 5 usually provides input to higher order thalamic nuclei, via axon collaterals of subcortical projections targeting the superior colliculus and spinal cord, amongst others (Deschênes et al. 1994). These are powerful and large, feed-forward “driver” inputs for relay to other cortical areas (Rouiller and Welker 2000; Reichova and Sherman 2004; Groh et al. 2008).

Retrograde labeling studies targeting posteromedial and lateral posterior thalamic nuclei (Po and LP) already suggested that layer 6b might specifically target these nuclei from primary somatosensory cortex (S1; Killackey and Sherman 2003) and from primary visual cortex (V1; Roth et al., 2016), but the injection sites lacked the resolution to specifically suggest lamina-specific patterns. Using anterograde techniques, Bourassa et al. (1995) described that all cells of the lower part of primary somatosensory barrel field (S1BF) layer 6 project to Po, but the majority of them also collateralized in VPM, and that a minority of cortico-thalamic cells in the lower portion of layer 6, may be located in septa regions, arbourized exclusively in Po. Single cell transcriptomic approaches combined with retrograde labeling demonstrated the unique characteristics of layer 6b (Tasic et al. 2016; Chevée et al. 2018).

Distinguishing patterns specific to layers 5, 6a, and 6b has been delayed due to lack of layer-specific markers. Recently, transgenic mouse lines have become available to address some of the above issues. The Rbp4-Cre line expresses Cre-recombinase in some layer 5 corticofugal projection neurons (Gong et al. 2007); the Ntsr1-Cre line in the majority of layer 6a and some layer 6b cortico-thalamic neurons (Olsen et al. 2012; Chevée et al. 2018). In this study, we identified and characterized a Drd1a-Cre line that selectively labels a proportion of postnatal layer 6b neurons. Constructing the projectome from a subpopulation of layer 6b neurons in motor, somatosensory and visual cortex revealed that layer 6b projections preferentially target Po, LP, and other higher order and midline thalamic nuclei in the ipsi- and contralateral thalamus, as well as frontal and lateral association cortices. Single axon reconstructions confirmed that Drd1a-Cre positive layer 6b projections do not form side branches in (pre)-thalamic reticular nucleus (TRN). Both light and transmission electron microscopy revealed that these layer 6b projections have small boutons that form synapses in the higher order Po nucleus. This selectivity is an unexpected and novel discovery that suggests that there is a third type of cortico-thalamic projection in addition to layers 5 and layer 6a, from layer 6b.

## Methods

### Selection of Layer 6b Cre-Expressing Transgenic Mice for This Study

We initially considered several transgenic mouse lines, searching for selective labeling of layer 6b without layer 6a. The Golli-τ-eGFP mouse expresses tau-eGFP in layer 6a and 6b cells and neurites (Jacobs et al. 2007) and thus it does not distinguish between layer 6a and 6b projections (Piñon et al. 2009; Grant et al. 2016). The Lpar1-eGFP line (Hoerder-Suabedissen and Molnár 2013) provides excellent labeling of a selected subplate population, but there is an additional interneuron population at the layer 5-6 boundary that is labeled (Hoerder-Suabedissen and Molnár 2013; Pedraza et al. 2014; Marques-Smith et al. 2016), and the labeled neurites from these cells cannot be followed beyond the white matter, even with anti-GFP immunohistochemistry. CTGF-Tg2-Cre gives sparse L6b-specific Cre expression in adult brains (see Allen Brain Atlas: http://connectivity.brain-map.org/transgenic/experiment/100095120; last accesssed February 2018), but Cre is expressed more broadly during embryonic development, so that crossing with reporter strains such as Ai9 results in labeling of almost all projection neurons in the cortex (our unpublished results, and see Allen Brain Atlas). The Ntsr1-Cre line includes both layer 6a and 6b cortico-thalamic neurons (Olsen et al. 2012; Grant et al. 2016; Chevée et al. 2018). The Tg(Drd1a-cre)FK164Gsat/Mmucd (Drd1a-Cre) mice proved to be a reliable and largely selective line to monitor layer 6b projection neurons from early postnatal development onwards.

### Breeding and Maintenance of Transgenic Mice

All experiments on transgenic animals were performed in the animal facilities of the University of Oxford (UK) under a valid Animals (Scientific Procedures) Act project licence as well as with local ethical approval. Animals were maintained on a 12hr light/dark cycle with free access to food and water. Tg(Drd1a-cre)FK164Gsat/Mmucd (Drd1a-Cre) mice were obtained from Mutant Mouse Regional Resource Centre and crossed with B6;129S6-*Gt(ROSA)26Sortm14(CAG-tdTomato)Hze*/J (Ai14) mice. Additionally, to confirm overlap between populations of subplate cells labeled in different transgenic lines, Drd1a-Cre/+;Ai14 females were crossed with either Lpar1-eGFP (Hoerder-Suabedissen and Molnár 2013) or Golli-tau-eGFP (Jacobs et al. 2007) males.

For transmission electron microscopy (EM) and to determine synaptic morphologies following viral injections, L5 and L6a Cre-expressing strains were additionally used (Rbp4-Cre;Ai14 and Ntsr1-Cre;Ai14 respectively (Gong et al. 2007; Olsen et al. 2012).

For biocytin tracing, experiments were performed on twelve 57BL/6 adult male mice, weighing between 25–30 g. All animal procedures on wild-type mice were approved by the Ethics Committee of the Autonoma University of Madrid, in accordance with European Community Council Directives (2010/63/UE) (Table 1).

**Table 1:** Summary table of co-expression of other subplate and cell-type markers in Drd1a-Cre;tdTom^+^ cells

	% of Drd1a-Cre;tdTom^+^ cells expressing other marker (mean ± SEM)	Number of brains	Number of sections /brain	Age	Region
NeuN	All	2	3 per region	5 months	S1 and M1
NeuN	93% ± 4	4	3	P8	S1
CTGF	34% ± 17	4	3	P8	S1
Cplx3	31% ± 8	4	3	P8	S1
Neuroserpin	29% ± 5	4	3	P8	S1
Lpar1-eGFP	None	2		P8, P21	
Golli-tau-eGFP	Most	2		P8, P24	

### Cre-Dependent Adeno-Associated Virus Injection into Targeted Brain Regions

To trace the axonal projections of Cre^+^ subplate cells, especially to subcortical targets, AAV2-CAG-Flex-ArchT-GFP (University of North Carolina Vector core) was injected in motor (MO), somatosensory (SS), SS/visual (SS/VIS) or visual (VIS) cortex of Drd1a-Cre/+;Ai14 adult animals (age range 6 weeks–4 months), or SS of Ntsr1-Cre;Ai14 or Rbp4-Cre;Ai14 mice (age range 7 weeks–4 months). Mice were deeply anaesthetized with isoflurane, and placed in a stereotaxic frame. Following midline skin-incision, craniotomies were performed over target cortex. Virus-filled pulled glass-micropipettes were inserted into the brain to the required depth (see Supplementary Table 1) and 200 nL of virus were slowly pressure ejected. Pipettes were retracted 5 min after the last ejection of virus, followed by postsurgery repair and recovery of the animal. Postinjection survival ranged from 3 to 4 weeks. See Supplementary Table 1 for target region and number of animals injected for each strain.

### Tissue Preparation for Immunohistochemistry and Fluorescence Axon Tracing

Cre^+^;Ai14+ brains ranging in age from embryonic day (E)15 to adult were collected for analysis of Cre expression pattern and distribution of projections. Embryonic brains were obtained from timed-pregnant females killed by Schedule 1 cervical dislocation. The uterus was removed and individual embryos dissected out in ice-cold 0.1 M phosphate buffered saline (PBS). Embryos were decapitated and heads were immersion fixed in 4% formaldehyde solution (FA, Sigma-Aldrich F8775) in 0.1 M PBS for 24 h. Postnatal mice were anesthetized with pentobarbitone (150 mg/kg, Pentoject, Animal Care Ltd, UK) and transcardially perfused with 0.1 M PBS followed by 4% FA. Brains were dissected out of the skull and postfixed in 4% FA for 24hrs. All tissue was stored in 0.1 M PBS with 0.1% sodium azide for long-term storage. Brains were embedded in 4.5% agarose (Bioline) in PBS and cut coronally to 50 μm on a vibrating microtome (Leica VT1000S). For serial two-photon imaging (see below), brains were embedded in 4% oxidized agarose and cross-linked with a 0.2% sodium borohydrate solution (in 0.05 M sodium borate buffer, pH 9.0–9.5).

### Immunohistochemistry

Unless otherwise stated, tissue was selected to include the primary somatosensory cortex (S1). Sections were blocked and permeabilized with 2% donkey serum and 0.1% Triton-X100 in 0.1 M PBS for 2 h at room temperature (RT). Primary antibody in blocking solution was applied overnight at 4°C. Following 3 washes, secondary antibody (diluted 1:500 in block) was applied for 2 h at RT. Sections were washed, counterstained with DAPI, mounted and coverslipped in PBS before being imaged on an epifluorescence microscope or laser scanning confocal microscope. The following primary antibodies were used: anti-neuroserpin, anti-connective tissue growth factor (CTGF), anti-complexin 3 (Cplx3), anti-vesicular glutamate transporter 1 (VGluT1), or anti-red fluorescent protein (RFP). The following secondary antibodies were used: donkey antimouse-AlexaFluor488 (1:500), donkey-antirabbit-AlexaFluor488 (1:500), and donkey antigoat-AlexaFluor488 (1:500), donkey antirabbit-AlexaFluor568 (1:500) as well as biotinylated donkey-antiguinea-pig or donkey-antimouse (1:100) (for summary see Supplementary Table 2). Cell counts were done in at least 2 brains per age and antibody, from several sections (typically 3) per brain. Results are given as mean ± SEM.

### Anterograde Labeling of Single Neurons with Biotinylated Dextran Amine

To anterogradely label L6b, L6a, or L5b cortico-thalamic axons, lysine-fixable biotinylated dextran amine (BDA), 10 000 MW (Invitrogen, CA, USA) was stereotaxically iontophoresed into layer 6 (or 5b) of S1BF (AP: −0.9 mm, L:3 mm, DV: −1.35 mm (for L6b), −1.0 mm (for L6a), or −0.8 mm (for L5b)). Animals were anesthetized with an intraperitoneal injection of a mixture of ketamine (Imalgène 500; 79 mg/kg) and xylazine (Rompun; 16 mg/kg). Then, animals were positioned in a stereotaxic apparatus, and maintained under isofluorane anesthesia. The sagittal midline of the scalp was sectioned and retracted, and a small craniotomy was drilled over S1BF. A micropipette (outer tip diameter 4–5 μm) containing 3% BDA in 0.01 M pH 7.4 phosphate buffer (PB) was stereotaxically positioned. Current pulses (100–150 nA, 1 s on/off) were delivered for 40–50 min through an iontophoresis device (PS-100 Microiontophoresis Dual Current 260, World Precision Instruments, Florida, USA).

After a survival period of 7 days, the animals were overdosed with pentobarbital (80 mg/kg, i.p) and perfused transcardially with 50 mL of saline followed by 100 mL of 4% paraformaldehyde (FA) in 0.1 M PB (pH7.4). The brains were postfixed in PFA for 4–6 h and cryoprotected in sucrose solution (30%) for 24 h at 4°C. Coronal 50 μm-thick sections were obtained on a freezing microtome (Leica SM 2400). BDA was visualized using a nickel sulfate enhanced glucose-oxidase (Shu et al. 1988) and an avidin-biotin-peroxidase (ABC; Vector, CA, USA) histochemical protocol. Subsequently, the free-floating sections underwent cytochrome oxidase histochemistry (Wong-Riley et al. 1978) for anatomical reference. Sections were finally mounted on glass slides, dehydrated, defatted in xylene and coverslipped with DePeX mounting medium (Serva, Heidelberg, Germany).

### Electron Microscopy Sample Preparation and Image Acquisition

Postembedding immunoelectron microscopy was performed on Drd1a-Cre::tdTom or Rbp4-Cre::tdTom axons in dissected Po. Deeply anaesthetized mice were perfused transcardially with 15 mL of fixative containing 4% FA and 0.2% glutaraldehyde in PBS. Dissected brains were postfixed in the same fixative overnight at 4°C and cut serially in 80 μm-thick coronal sections on a vibrating microtome (Leica VT1000S). Sections containing Po were selected and the Po region was dissected out under a fluorescence microscope (Leica MZFLIII). After staining with 2% uranyl acetate in 0.1 M sodium acetate buffer for 45 min–1 hr, the tissues were dehydrated through a graded series of methyl alcohol (70%, 90%, and 100%) and embedded in LR gold resin (Agar Scientific) containing 0.5% benzil (Agar Scientific). The resin was polymerized under UV light for 16–18 h at −20°C. Ultra-thin sections were prepared and mounted on formvar-coated 200-mesh nickel grids (Agar Scientific). For immunogold labeling for tdTomato, sections were blocked with 1% chicken egg albumin (Sigma-Aldrich) in PBS and incubated with rabbit anti-red fluorescent protein (RFP) antibody (1:500, MBL international) for 2 h and then with 20 nm gold particle-conjugated goat antirabbit (1:50, BBI solutions) for 1 h. For detection of VGluT1, anti-VGluT1 (1:500, Millipore) and 10 nm gold particle-conjugated goat anti-Guinea pig (1:50, BBI solutions) were used. After postfixation with 1% glutaraldehyde for 10 min, sections were lightly counterstained with uranyl acetate and lead nitrate. All antibodies were diluted in the blocking solution. Sections were examined on a JEOL 1010 transmission electron microscope (JEOL) with 3000–12 000× magnification. To quantify the size of Drd1a-Cre::tdTom^+^ and Rbp4-Cre::tdTom^+^ axon terminals in Po, immunogold labeled boutons forming a synapse in single sections were randomly selected and the cross-sectional area of those boutons was measured with ImageJ software (http://imagej.nih.gov/ij/). To obtain the net size of the Rbp4-Cre::tdTom^+^ boutons, the areas of excrescences invading the boutons were excluded. Data (from *n* = 20 synapses in Po from 2 Drd1a-Cre/+;Ai14 brains and *n* = 30 synapses from 2 Rbp4-Cre/+;Ai14 brains) were analyzed by one-way ANOVA followed by Dunn’s Multiple Comparison Test using GraphPad Prism 4 software

### Imaging and Analysis

Distribution of Drd1a-Cre::tdTom labeled cells and fibers from the whole cortical mantle was documented using a Leica epifluorescence microscope (DMR) equipped with a Leica DC500 CCD camera. Images or tiled-image stacks of antibody stained sections to demonstrate co-localization (or lack thereof), and to visualize synaptic terminals in virus injected brains were acquired with a laser scanning confocal microscope (Zeiss LSM710). All results are given as mean ± SEM.

For BDA-labeled material, sections were systematically examined under brightfield optics using a Nikon Eclipse E80i microscope, and the layer location of the BDA injection was determined. The labeled terminal fields were examined and images taken using a microscope camera (DMX 1200F, Nikon). The cell bodies and entire axonal trajectories of BDA labeled neurons were drawn from the serial sections using camera lucida.

Co-localization with other subplate markers was quantified from confocal image stacks taken in S1 cortex. *N* = 4 P8 Drd1a-Cre/+;Ai14 brains per antibody were used, and *n* = 2 adult Drd1a-Cre/+;Ai14 brains for NeuN.

Varicosities were also measured in BDA-labeled axons in Po originating from L5b, L6a, or L6b cells. As above, for each condition, 3 experimental cases were measured (80–100 varicosities per experimental sample) and the results averaged. Labeled profiles were identified as varicosities when their diameter was at least twice that of the adjacent axonal segments. We used Nis-Elements® BR3.2 software (Nikon) to manually draw the varicosity contour and calculate its maximal projection (cross-sectional) area. We did not include varicosities with cross-sectional areas near the optical microscope resolution limit (<0.2 μm^2^). Varicosity sizes were grouped in discrete intervals of 0.5 μm^2^ and these distributions compared between cells in each cortical layer (two-sample Kolmogorov–Smirnov, K–S, test). Mean sizes were also directly compared with non-parametric Mann–Whitney *U* test. Differences were considered significant when *P* < 0.05. Bouton size was also measured on single image planes of transmission electron micrographs.

The 3D projection pattern of adeno-associated virus (AAV) injected brains was examined using serial two-photon tomography. The serial two-photon (TissueCyte 1000; Tissuevision) imaging procedure used has been previously described in detail (Ragan et al. 2012; Kim et al. 2015, 2017; Jeong et al. 2016). Briefly, the fixed brain was embedded and cross-linked in the oxydized agarose. The entire brain was imaged as 12 × 16 × 280 (*xyz*) tiles with a 1 × 1 μm^2^*xy* resolution for every 50 μm *z*. A wavelength of 910 nm was used for two-photon excitation to excite both red and green fluorophores. Red and green emission spectra were separated using a 560 nm dichroic mirror (Chroma, T560LPXR) and band pass filters (Semrock FF01-607/70-25 for red and FF01-520/35- 25 for green signal). The resulting tiles in each channel were stitched with custom-built software, and 3D rendering was done using the Volocity software (Perkin Elmer). Data processing for anterograde projections have been described in detail previously (Jeong et al. 2016). Briefly, signal to noise ratio was enhanced by using the “subtract background” function in FIJI (ImageJ, NIH) with rolling ball radius = 10. Then, the signal was converted into the binary signal by applying the “Threshold” function in FIJI. Then, the number of signal pixels in a 20 × 20 pixel (1pix = 1 μm) evenly spaced rectangular box was calculated and used as projection density at a given area. The projection signal from each brain was registered in the Common Coordinated Framework from Allen Brain Institute and projection signals from different brains were pseudo-colored to facilitate comparison in the same reference brain.

## Results

Subplate cells are a diverse population of cells, many of which are lost during early postnatal development, but some survive as layer 6b cells forming a thin layer of cells between the white matter and layer 6a in the adult mouse brain (Hoerder-Suabedissen and Molnár 2015). Here we describe a transgenic mouse strain (Tg(Drd1a-cre)FK164Gsat/Mmucd; here referred to as Drd1a-Cre) that labels a substantial subpopulation of mouse layer 6b cells. This is the first, relatively selective tool available to distinguish pure layer 6b projections from the other cortical layers at the regional level, and has been instrumental in revealing regional differences in the layer 6b projectome.

### Development of Cre-Expression in the Brain of Drd1a-Cre; Ai14 Mice

**Figure 1.**
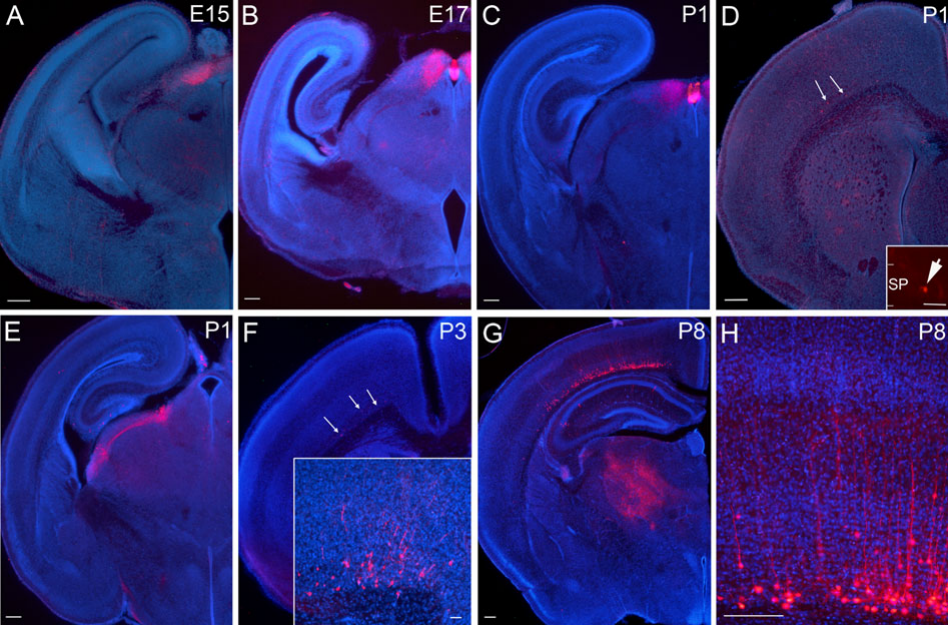
**Drd1a-Cre expression in the brain visualized by tdTomato labeling in Drd1a-Cre; Ai14 mouse from embryonic development until P8**. (*A*,*B*) At E15 and E17, Cre-expressing cells are restricted to a small area of the dorsal thalamus, no Cre^+^ cells are found in the cerebral cortex. In caudal sections the labeled regions are in contact with the aqueduct, from where they continue anteriorly in a dorsal position. (*C*–*E*) At P1, Cre^+^ cells are most abundant in dorsal thalamus, but a few Cre^+^ cells are located in the subplate/layer 6b of the anterior medial cerebral cortex (see inset in *D*). (*F*) By P3, the cortical layer 6b cell population has expanded in number and extends to more lateral and posterior locations. Cells are also beginning to have a clear pyramidal morphology (see inset in *F*). (*G*–*H*) At P8, cortical Cre^+^ cells are abundant, with more cells located in medial and anterior cortex. Cells extend projections to higher order thalamic nuclei (*G*) and dendrites into the overlying cortex (*H*). Scale bars = 200 μm (A-H) and 50 μm (all insets).

To examine the Cre-expression, we crossed the Drd1a-Cre line with Ai14 mice and studied the tdTomato expression. At embryonic day (E)15 and 17, tdTomato-labeled (tdTom^+^) cells are restricted to the preoptic tectum or superior colliculus and in a restricted area at the dorsal edge of the aqueduct in the midbrain (Fig. 1*A*,*B*). Labeled cells were not observed in any other brain structure.

During the first postnatal week, labeled cells in subplate/L6b are first observed. At P1, the first cells are visible in medial, anterior cortex (Fig. 1*D*). The density of labeled cells is higher at P3, and extends both more laterally and more caudally than at P1 (Fig. 1*F*). The labeled cells at P3 also have a more defined morphology, compared to P1 when dendrites are very short. In motor cortex (MO) at P3, based on their dendrites and soma shape, the tdTom^+^ cells resemble the “pyramidal-like”, “multipolar”, and”tangential” morphologies described by Marx and colleagues, however there are hardly any tdTom^+^ cells in S1 that would permit classification in the same region as Marx et al. (2017). Most cells have a very prominent (fat and short) dendrite, but it is not always oriented towards the pial surface. Abundant tdTom^+^ cells remain in the vicinity of the aqueduct in a small region posterior to the optic tectum, as observed at younger ages. Cells are additionally present in 2 distinct dorsal thalamic nuclei, with fiber bundles extending between them (Fig. 1*E*).

In P8 brains (Fig. 1*G*) tdTom^+^ cell distribution is similar to that observed at all subsequent ages, but projections are less well developed at this stage. The density of cells in the subplate/L6b increases substantially between P3 and P8. For quantification, P3 and P8 brains in the region of motor cortex (MO) were labeled with anti-RFP antibody, and cells counted in the subplate up to 80 μm above the white matter (WM)-gray matter boundary (887 ± 68 cells/mm^2^ at P3 (*n* = 2 brains) compared to 3793 ± 291 cells/mm^2^ at P8 (*n* = 3 brains)). From P8 and onwards, tdTom^+^ cells in most cortical areas are too dense to be able to reconstruct full dendritic arbors, but most cells appear pyramidal shaped, with a prominent apical dendrite. Multipolar neurons are also quite common, especially in more medial regions. Some neurons, especially in somatosensory regions, are tangentially oriented (with a single, prominent dendrite).

From P8 onwards, cells are also present in deep layer 6a in more medial and anterior cortical regions, and first emerge in layer 6b in lateral somatosensory regions. At P8 and all subsequent ages imaged, very sparse cells are present in the hippocampus and striatum, and more densely in some midbrain nuclei and the cerebellum (see Supplementary Fig. 1 for adult data). At no time point is labeling observed in the eye or retina, nor in the olfactory bulbs. More details of later developmental stages, as well as a description of non-neuronal Cre-expression can be found in the Supplementary materials (Supplementary Fig. 1 and Supplementary Table 3).

In adult brains, GFP-labeling of tdTom^+^ cells is obtained following injection of Cre-dependent AAV, suggesting enduring and consistent Cre-recombinase expression into adulthood in the cortex (see below).

### Drd1a-Cre Labeled Cells Are a Subpopulation of Subplate Neurons

**Figure 2.**
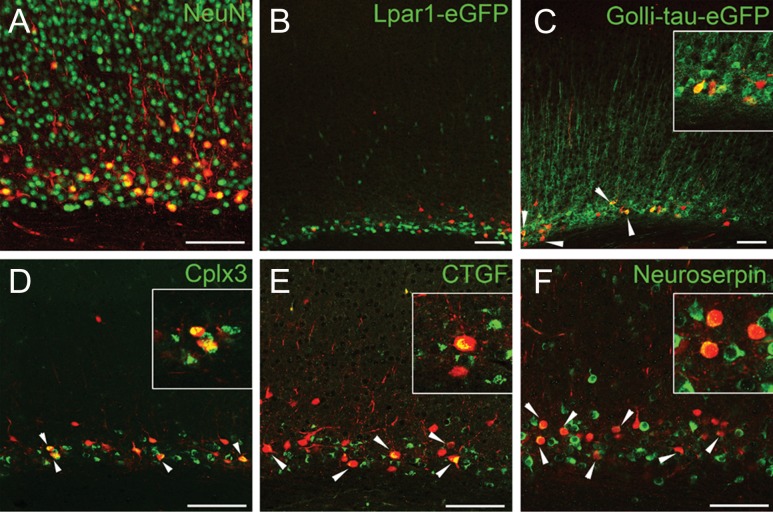
**Drd1a-Cre::tdTom^+^ neurons co-express Cplx3, CTGF and Neuroserpin, but do not co-localize with Lpar1-eGFP at P8**. (*A*) Drd1a-Cre::tdTom^+^ cells are NeuN^+^ neurons, and co-express Cplx3 (*D*), CTGF (*E*) and Neuroserpin (*F*; arrowheads). Drd1a-Cre::tdTom^+^ cells overlap with Golli-tau-eGFP^+^ cells in triple transgenic brains (*C*, arrowheads). Drd1a-Cre::tdTom^+^ cells do not overlap with Lpar1-eGFP^+^ cells in triple transgenic brains (*B*). All images are from primary somatosensory cortex. Insets show examples of co-localization at higher magnification. Scale bars = 100 μm.

Postnatal subplate neurons can be divided into multiple subpopulations depending on marker expression (Hoerder-Suabedissen et al. 2009; Hoerder-Suabedissen and Molnár 2013; Tasic et al. 2016) and functional connectivity (Meng et al. 2014). We first aimed to determine which subpopulation is labeled in this line. To establish what proportion of the subplate neurons were labeled in Drd1a-Cre::tdTom mice, we used antibodies to a neuronal specific marker, NeuN^+^ (Fig. 2*A*). Drd1a-Cre::tdTom cells are NeuN^+^ neurons, and make up approximately one-fifth of neurons in the subplate of somatosensory cortex at P8 (18% ± 7 of 547 NeuN^+^ cells were tdTom^+^; *n* = 3 brains and 3 sections per brain) or one quarter of neurons in the subplate of somatosensory cortex in adult brains (24% ± 4 of 284 NeuN^+^ cells were tdTom^+^; *n* = 2 brains and 3 sections per brain).

We examined the overlap of the Drd1a expression pattern with that observed in several other mouse lines in order to understand the characteristics of the Drd1a-positive population. Drd1a-Cre;Ai14 mice were crossed with Lpar1-eGFP males (see (Hoerder-Suabedissen and Molnár 2013) for a description of this strain). GFP^+^ offspring with tdTomato-labeled cortical cells were obtained at P8 and P24 (*n* = 1 brain at each age). No overlap was observed between tdTom^+^ and GFP^+^ cells within the subplate layer at either age (Fig. 2*B*). Drd1a-Cre;Ai14 mice were also crossed with Golli-τ-eGFP (GTE (Jacobs et al. 2007)) males. GFP^+^ offspring with tdTomato-labeled cortical cells were obtained at P8 and P21 (*n* = 1 at each age). Most tdTom^+^ cells within the subplate were also GFP^+^, similarly for cells located in layer 6a (Fig. 2*C*). However, many GFP^+^ cells were not tdTom^+^, suggesting that GTE labels a larger population of cells, especially in layer 6a and in more lateral, primary sensory areas.

Drd1a-Cre::tdTom^+^ cells co-express other proteins that are known to be expressed in subplate neurons such as Complexin 3 (Cplx3; Fig. 2*D*), Connective Tissue Growth Factor (CTGF; Fig. 2*E*) and Neuroserpin (Fig. 2*F*; Hoerder-Suabedissen et al. 2009; Viswanathan et al. 2012, 2016; Hoerder-Suabedissen and Molnár 2013; Kondo et al. 2015). In S1, approximately one-third of P8 tdTom^+^ subplate cells each are CTGF^+^, Neuroserpin^+^ or Cplx3^+^ (34% ± 17 of 107 Drd1a-Cre::tdTom^+^ cells were CTGF^+^; 29% ± 5 of 85 Drd1a-Cre::tdTom^+^ cells were Neuroserpin^+^; 31% ± 8 of 112 Drd1a-Cre::tdTom^+^ cells were Cplx3^+^). In wild-type mice, there is partial overlap between CTGF and Cplx3 (Hoerder-Suabedissen and Molnár 2013) and between Neuroserpin and CTGF or Cplx3 (data not shown). Drd1a-Cre::tdTom is present in roughly one-tenth of the CTGF^+^, Cplx3^+^ or Neuroserpin^+^ cell populations in S1. Specifically, 10% ± 6 of 320 CTGF^+^ cells were Drd1a-Cre::tdTom^+^, 10% ± 2 of 310 Cplx3^+^ cells were Drd1a-Cre::tdTom^+^ and 6% ± 2 of 351 Neuroserpin^+^ cells were Drd1a-Cre::tdTom^+^ at P8, in S1 (*n* = 3-4 P8 brains per antibody combination).

### Projections from Drd1a-Cre::tdTom^+^ Neurons Project to the Thalamus by P6

We examined the development of neurites extending from the Drd1a-Cre::tTom^+^ cells. No axons derived from cortical Drd1a-Cre::tTom^+^ cells are traceable in neonatal brains, and short primary dendrites ascend vertically into overlying cortical structures by P3 (Fig. 1*F*). No descending axons are visible in the striatum or internal capsule at this age, even if the native tdTomato fluorescence is boosted by anti-RFP immunohistochemistry. By P8, Drd1a-Cre::tTom^+^ cells have long, and mostly unbranched dendrites extending into L5 (Fig. 1*H*), and axons are visible in the thalamus (Fig. 1*G*). tdTom^+^ fibers are detectable in the marginal zone (although most branching occurs at the upper edge of L5) and in bundles in the white matter and entering the striatum. Sparse and branched fibers are evident in some thalamic nuclei at P8 (Fig. 1*G*). Sparse, tdTom^+^ puncta can be visualized within the LGd and VPM, with denser fiber plexus developing in Po. At P6, cells located in the subplate can be backlabeled with DiA placed into Po thalamus, and some of these cells are tdTomato^+^ (*n* = 1 brain at P6; Supplementary Fig. 2).

**Figure 3.**
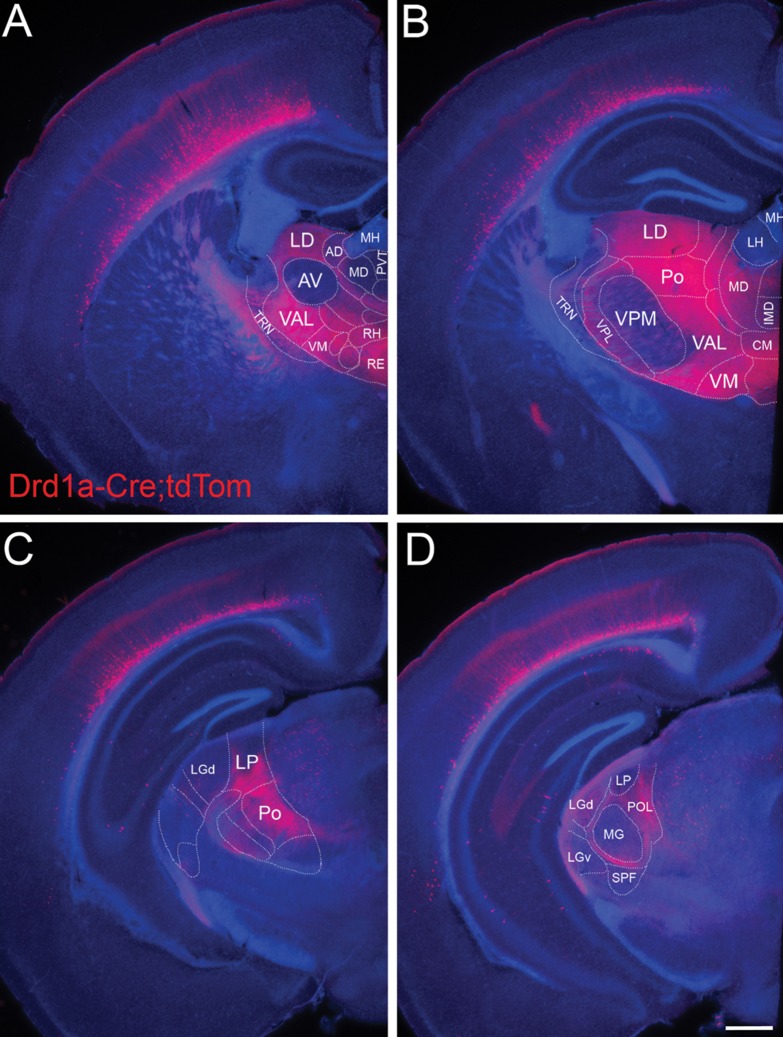
**Projections from Drd1a-Cre::tdTom L6b neurons target second-order and avoid first-order thalamic nuclei**. In adult (P35) brains, labeling of Drd1a-Cre::tdTom^+^ fibers derived from the entire cortical mantle reveals that layer 6b neurons target higher order thalamic nuclei while avoiding relay thalamic nuclei. (*A*) Projections from layer 6b avoid anterior ventral nucleus (AV) as well as the habenula and paraventricular thalamic nucleus, whereas higher order nuclei such as lateral dorsal (LD) or ventral anterior lateral (VAL) and ventral medial (VM) are densely filled with terminal branches. Note that within cortex, the majority of processes from L6b end below L4 in barrel cortex, except for a dense band of branched fibers in the marginal zone. (*B*) Layer 6b projections also largely avoid ventral-posterior-medial (VPM) but densely branch in posterior nucleus (Po). Fibers pass through TRN and ventral-posterior lateral nucleus (VPL) without apparent branching. Some midline nuclei such as central medial (CM) are innervated whereas others are not. (*C*) Fibers from layer 6b also avoid the first-order visual thalamic nucleus (lateral geniculate nucleus—LGd) anteriorly but target the higher order visual nucleus lateral posterior (LP), and a small region of posterior LGd (*D*). (*D*) Fibers from layer 6b bypass the first-order auditory nucleus (medial geniculate nucleus) in the thalamus. Note the scattered tdTom^+^ cells in the midbrain, including superior colliculus and anterior pretectal nucleus visible at this level. Scale bar = 500 μm. AD, anterodorsal nucleus; AV, anteroventral nucleus; CM, central medial nucleus; IMD, intermediodorsal nucleus; LD, lateral dorsal nucleus; LH, lateral habenula; LGd, dorsal lateral geniculate nucleus; LGv, ventral lateral geniculate nucleus; LP, lateral posterior nucleus; MD, mediodorsal nucleus; MG, medial geniculate nucleus; MH, medial habenula; Po, posterior nucleus; POL, posterior limiting nucleus; PVT, paraventricular nucleus; RE, nucleus reuniens; RH, rhomboid nulceus; SPF, subparafascicular nucleus; TRN, (pre-)thalamic reticular nucleus; VAL, ventral anterior lateral complex; VM, ventral medial nucleus; VPL, ventral-posterior lateral nucleus; VPM, ventral-posterior medial nucleus.

In P21, P35, and adult brains, cortical Drd1a-Cre::tTom^+^ cells extend labeled processes into a variety of cortical and subcortical brain structures (Fig. 3). Within the neocortex, labeled dendrites from L6b cells terminate well below L4, whereas dendrites from some L6a cells extend into supragranular layers. Labeled axons form a dense network in the marginal zone and another dense network at the border between L5 and L4, sometimes extending into the septa between barrels in S1, as has been previously reported for Cplx3^+^ layer 6b cells (Viswanathan et al. 2016) and also for the mixed layer 6a and 6b-derived neurites labeled in the Golli-τ-eGFP and Ntsr1-Cre mice (Piñon et al. 2009; Grant et al. 2016).

No Drd1a-Cre::tTom^+^ axons are visible in the corpus callosum (Fig. 3*A*) or anterior commissure (data not shown). Dense axon bundles course within the white matter, and descend into the striatum and internal capsule. Subcortically, labeled axons, densely branch in higher order thalamic nuclei (lateral dorsal (LD), ventral anterior lateral (VAL), ventral medial (VM), and Po nucleus; Fig. 3*A*–*C*), and many of the non-specific thalamic nuclei near the midline including rhomboid (RH) and reuniens (RE), medial dorsal (MD), and central medial (CM), amongst others. First-order, or relay thalamic nuclei such as ventral posteriolateral (VPL, Fig. 3*B*), most of LGd (Fig. 3*C*), medial geniculate nucleus (MGN, Fig. 3*D*), or the anterior ventral nucleus (Fig. 3*A*), which “relays” to cortex input from the basal ganglia, receive sparse, thin, branched fibers, but the innervation is much less dense than for higher order nuclei. However, posterior LGd (Fig. 3*D*) receives more robust input. Similarly sparse innervation is found in the anterodorsal (AD) nucleus. Fibers are completely absent from paraventricular thalamic nucleus and the habenular nuclei. No side branches are formed in the TRN (Fig. 3*A*,*B* and Supplementary Fig. 3*B*), but unbranched fibers traverse this region en route to more medial thalamic structures. Thick fiber bundles without side branches are also evident in VPM.

Additionally, labeled fibers are present in posterior optic tract. These are not derived from the eye, as there are no Drd1a-Cre::tTom^+^ cells in the retina or other parts of the eye (*n* = 2 Drd1a-Cre/+;Ai14+ animals aged P21, and *n* = 2 adult males). The superior colliculus also receives dense innervation of Drd1a-Cre::tTom^+^ fibers, arriving via the optic tract. The midbrain contains Cre-expressing cells, but no dense areas of fiber terminations (Fig. 3C, Supplementary Fig. 1).

### Distribution and Morphology of Projections in Higher Order Thalamic Nuclei from Identified Cortical Areas

Previous studies using retrograde labeling from Po revealed backlabeled neurons in layers 6b and 5 suggesting that this thalamic nucleus is innervated by neurons in both of these cortical layers (Killackey and Sherman 2003; Chevée et al. 2018), and injections of retrograde tracers into lateral posterior nucleus of the thalamus (LP) label layer 6b neurons in visual cortex (Roth et al. 2016). However, retrograde labeling studies have limitations in their resolution of lamina-specific patterns.

**Figure 4:**
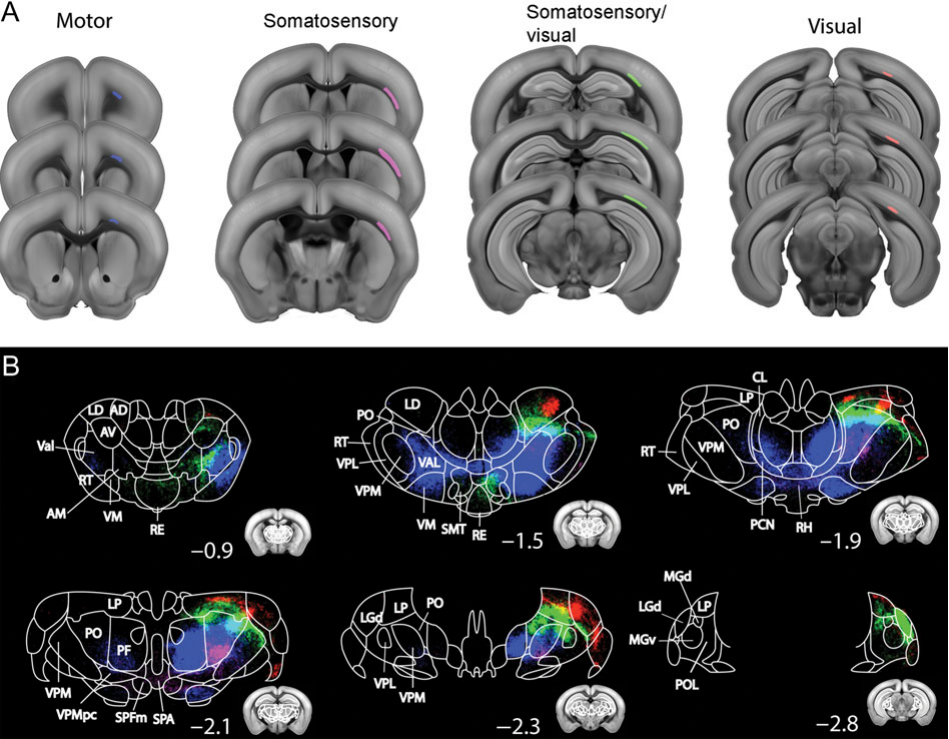
Projections from Drd1a-Cre^+^ layer 6b neurons from motor (MO), somatosensory (SS), SS/visual (SS/VIS), and visual (VIS) cortex target distinct regions of thalamus. Cortico-thalamic projections of Drd1-Cre from somatosensory and visual cortices using Cre-dependent AAV-eGFP tracing. (*A*) AAV infected areas targeting MO (blue), SS (magenta), SS/VIS (green) and VIS (red) cortex. (*B*) Thalamic projectome derived from the 4 cortical areas pseudo-colored with different colors matching the injection site (*A*). Small inserts represent the outline of the coronal sections; numbers indicate distance from bregma. Note, projections from different cortical areas have topographically segregated projections to mostly higher order thalamic areas. Some projections innervate the contralateral thalamic nuclei such as rhomboid (RH), reuniens (RE), submedial (SMT) and subparafascicular nucleus (SPF) as well as subparafasciular area (SPA) of the thalamus. AD, anterodorsal nucleus; AM, anteromedial nucleus; AV, anteroventral nucleus; CL, central lateral nucleus; LD, lateral dorsal nucleus; LGd, dorsal lateral geniculate nucleus; LP, lateral posterior nucleus; MGd, dorsal medial geniculate nucleus; MGv, ventral medial geniculate nucleus; PCN, paracentral nucleus; PF, parafascicular nucleus; PO, posterior nucleus; POL, posterior limiting nucleus; RE, nucleus reuniens; RH, rhomboid nulceus; SPA, subparafascicular area; SPF, subparafascicular nucleus; RT, (pre-)thalamic reticular nucleus; SMT, submedial nucleus; Val, ventral anterior lateral complex; VM, ventral medial nucleus; VPL, ventral-posterior lateral nucleus; VPM, ventral-posterior medial nucleus.

To label projections from layer 6b neurons of a defined cortical region, we injected Cre-dependent GFP-expressing AAV2 into adult Drd1a-Cre/+ or Drd1a-Cre/+;Ai14 mice (MO, *n* = 4; SS, *n* = 5; SS/VIS *n* = 3, VIS *n* = 3, Fig. 4). The brains were imaged by serial two-photon tomography and were registered to a reference brain atlas to examine the brain-wide projection pattern in a common anatomical space (see Methods). The axonal projection pattern, called “projectome”, was established for all 4 anatomical locations (see Fig. 4 and Supplementary Fig. 4). Due to the widespread of the virus after injection, all injection sites are likely to include small portions of adjacent other cortical areas (e.g., SS may include a small region of some motor associated areas).

We chose one representative sample from each injection area (motor, somatosensory, somatosensory/visual, visual), and compared their thalamic projectome (Fig. 4). GFP^+^ fibers from motor and somatosensory cortex descend into the white matter and then fasiculate in caudate putamen and internal capsule and traverse TRN. Those from motor cortex fasciculate lateral to VPL and course ventrally around it to reach more medial structures, whereas axons from somatosensory regions cross VPL and VPM in tight bundles in their respective dorsal halves. Axons from visual areas follow the internal capsule to reach TRN and then also traverse VPL and VPM in their dorsal halves.

Injections into all 4 cortical regions gave partially overlapping regions of dense branching and axon terminations in higher order and midline thalamic nuclei. Axons from the motor cortex densely project to medial anterior thalamus. In particular, axonal termination fields (in rostro-caudal order) are formed in VAL, VM, ventrolateral (VL) and CM thalamic nucleus, and extend to Po and the more medial parafascicular thalamic nucleus (PF) in caudal thalamus (blue in Fig. 4). Within thalamus, the majority of the termination fields target thalamic nuclei in both hemispheres, with the exception of Po, which appears only innervated on the ipsilateral side. However, for bilaterally innervated nuclei, the bulk of the terminations are still on the ipsilateral side. Mid-line crossing occurs at the level of RH and possibly also CM thalamic nuclei.

Projections from SS form the densest region of branching and axon terminals in Po, especially the ventral half (magenta in Fig. 4*B*). Sparser projections are present in VPM, especially near the boundary with Po, in RH, subparafascicular nucleus (SPF) and other ventral thalamic nuclei as well as the hypothalamus.

Projections from VIS form the densest region of branching and axon terminals in lateral dorsal (LD) nucleus of the anterior thalamus and LP and LGd (at the medial edge with LP) in caudal thalamus (red in Fig. 4*B*). The fibers in LP appear primarily derived from a dense fiber bundle traversing between VPM and LGd. Projections to LD and LP cover less than a quarter of the nucleus, in contrast to projections from MO which cover the majority of the respective nuclei that they target. The majority of subcortical projections from either restricted somatosensory or restricted visual areas are confined to the ipsilateral side. However, some midline crossing and contralateral arbourization is visible in RH, reuniens (RE), submedial (SMT) and SPF as well as subparafascicular area (SPA) of the thalamus (Fig. 4*B* and Supplementary Fig. 1).

The large injections covering caudal somatosensory and rostral visual cortical areas (SS/VIS) and the posterior parietal association regions (PTLp) give rise to terminations in the same nuclei as more selective sensory or visual injections, but more dorsal than anterior somatosensory and more ventral than most caudal visual derived projections. Target areas include Po and LP (green in Fig. 4*B*). Additionally, there are more bilateral projections, especially to the midline reuniens nucleus from this mixed somatosensory and visual injection.

Within thalamus, layer 6b projections show topographical segregation with partial overlap based on the injection sites (Fig. 4*B*). For example, anterior and posterior cortical injections target medial to lateral domains of the thalamus. The majority of subcortical projections from SS, SS/VIS or VIS areas are confined to the ipsilateral side, with contralateral arbourization in mid-line thalamic nuclei including RH, RE, submedial (SMT) and SPF nucleus as well as SPA. In contrast, projections from motor cortex show strong contralateral projections including contralateral VAL (Fig. 4B, Supplementary Fig. 4).

Within thalamus, layer 6b projections from the rostral somatosensory area target more ventral areas of Po compared with projections from the caudal somatosensory/rostral visual injection (Fig. 4B).

Since cortical thalamic projections are also derived from other deep layers (e.g., layer 5), we compared the projectome from L6b-specific Drd1-cre to other deep layer-specific Cre drivers (Rbp4-Cre for L5 and Ntsr1-Cre for L6a and b) projectome data (downloaded from Allen connectivity database, http://connectivity.brain-map.org/) from a matching somatosensory cortical area. In contrast to the projectome from Drd1-Cre mainly targeting higher order structures (e.g., Po), the projectome from Ntsr1-Cre showed similar innervation in both higher order (e.g., Po) and primary thalamic nuclei (e.g., VPM; Supplementary Fig. 5, as shown by the previous literature (Olsen et al. 2012; Grant et al. 2016). L5 specific Rbp4-Cre showed denser innervation in higher order thalamus with less in first-order thalamic nuclei (Supplementary Fig. 5, again in agreement with the previous literature reporting L5 as primarily targeting higher order thalamic nuclei (Deschênes et al. 1994; Grant et al. 2016). Thus, based on this region-specific projectome, the targeting of L6b thalamic innervation is more similar to that of L5, than L6a.

### Layer 6b Projections Do Not Form Side Branches in TRN

Layer 5 usually provides input to higher order thalamic nuclei, via axon collaterals of subcortical projections targeting the superior colliculus and spinal cord, among others, as described in rats (Deschênes et al. 1994). These are powerful and large, feed-forward “driver” inputs for relay to other cortical areas (Rouiller and Welker 2000; Reichova and Sherman 2004; Groh et al. 2008), with some interspecies variability in which cortical region provides the giant L5 terminals to a particular thalamic nucleus (Rouiller and Welker 2000). They may converge onto thalamic cells also receiving driver input from subcortical structures (Groh et al. 2014), but they do not form collaterals or synapses in TRN in mouse or rat (Bourassa et al. 1995; Rouiller and Welker 2000). In contrast, layer 6a cells form axon collaterals and synapses onto inhibitory neurons in TRN (Lam and Sherman 2010) and this has an impact on the frequency dependent modulation of thalamic function (Crandall et al. 2015).

**Figure 5.**
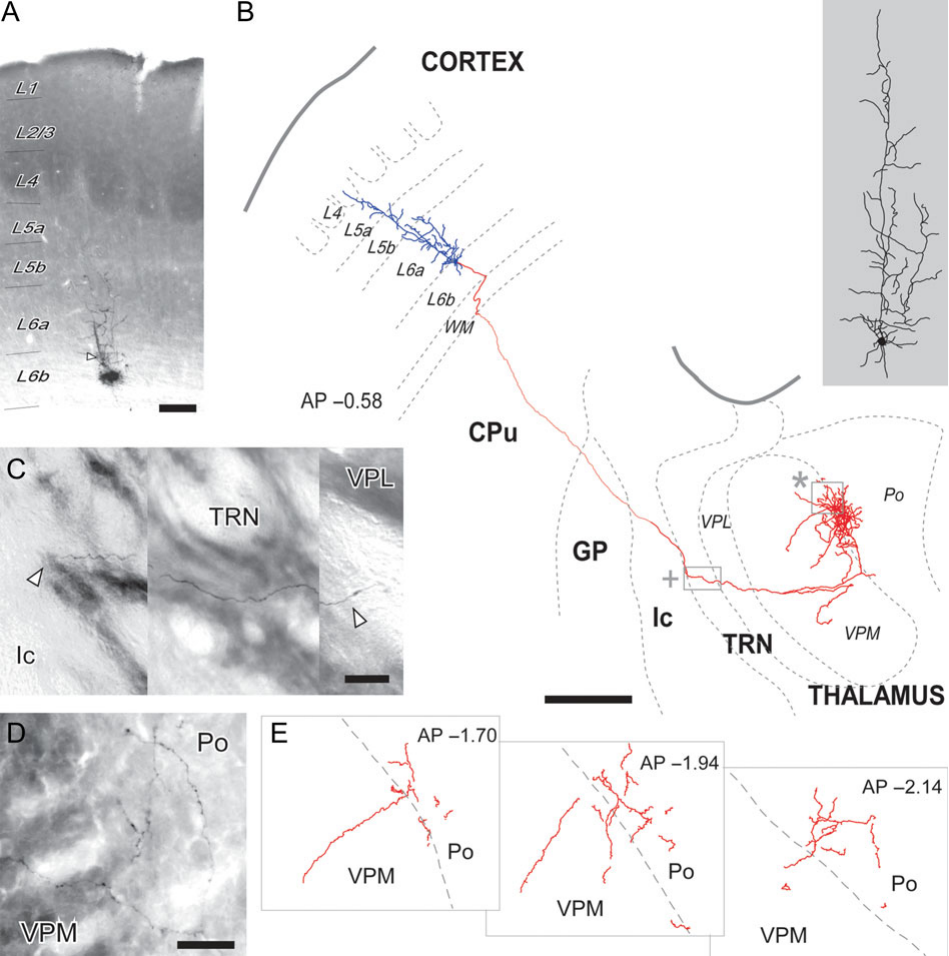
Single-cell labeling of a layer 6b neuron revealed the absence of side branches in TRN and dense innervation of lateral Po. (*A*) A coronal section of S1BF containing the microiontophoresis site in layer 6b (L6b). Cytochrome-oxidase counterstain revealed the barrels and Po and VPM boundary (not shown). The deposit center is visible as an amorphous black precipitate. Adjacent to it, the soma and dendrite of a labeled L6b cell (arrowhead) is visible. (*B*) Camera-lucida reconstruction of the somatodendritic arbor (blue) and axon (red) of the above L6b cell. The somatodendritic domain is drawn at higher magnification on the right (gray shaded area). Borders between cortical layers (L4-L6b) in cortex are delineated. AP, Distance to Bregma in the anteroposterior axis, in mm. CPu, caudate-putamen; GP, globus pallidus; Ic, internal capsule; TRN, reticular prethalamic nucleus; Po, posterior thalamic nucleus; VPL, ventral posterolateral thalamic nucleus; VPM, ventral posteromedial thalamic nucleus. (*C*) Photomontage showing the axon as it traverses TRN (inset in B marked by a cross). Arrowheads indicate the lateral and medial edges of Rt. (*D*–*E*) Details of the terminal axonal arbourization. In panel *D*, labeled segments are visible at the edge between Po and VPM (inset in B marked by an asterisk). Cytochrome-oxidase counterstain. Drawings in panel *E* show axon segments on several coronal levels. Scale bars: A = 150 μm; B = 500 μm; C = 25 μm; D = 50 μm.

**Figure 6.**
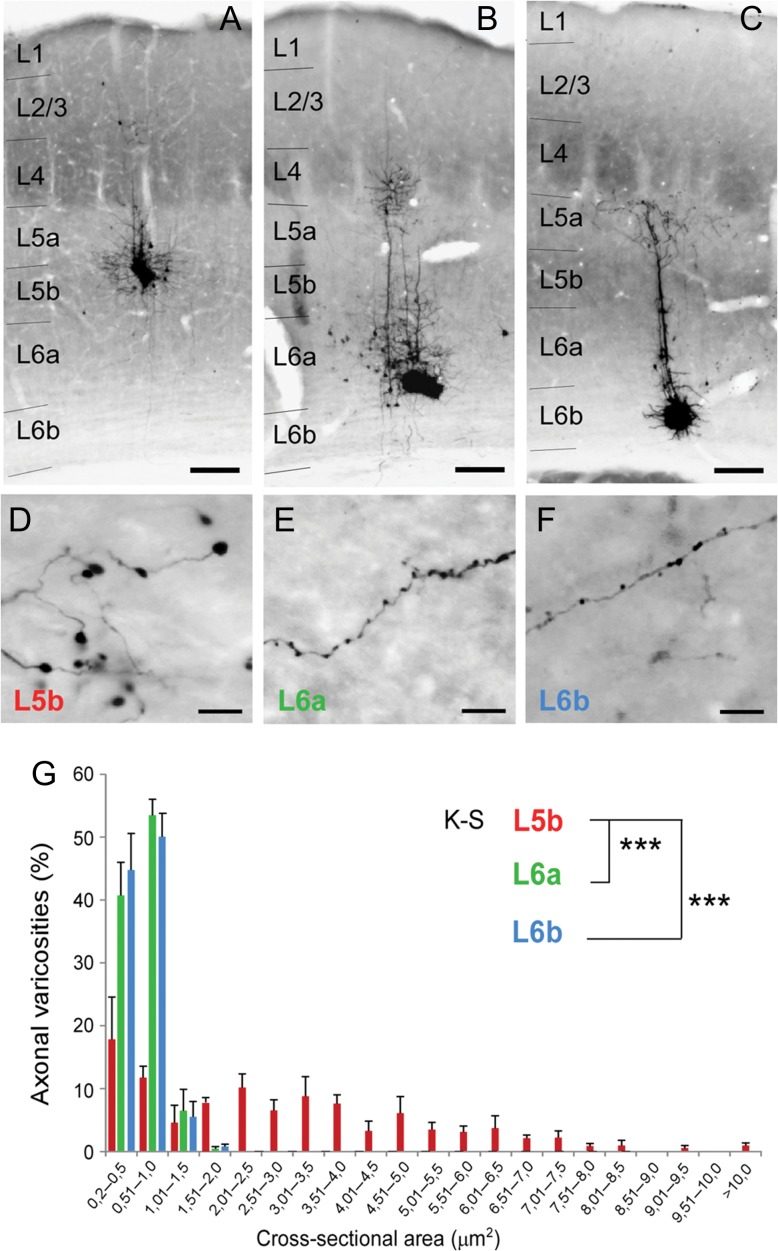
**L6b, 6a, and 5b cortical neurons have distinct morphologies of axonal varicosities in Po**. Photomicrographs showing BDA microdeposits in layer 5b (*A*), layer 6a (*B*), or layer 6b (*C*) in S1BF cortex, and details of their respective labeled axonal arbourizations in Po nucleus (*D*, *E*, and *F*). Cytochrome-oxidase histochemistry counterstain was used to delineate barrels in S1BF. (*G*) Size distribution of varicosities from L5b, L6a and L6b axons grouped into discrete intervals according to their maximal cross-sectional area. Scale bars: *A*–*C* = 150 μm, D-F = 10 μm.

To determine, whether layer 6b cells are more similar to L5 or L6a subcortical projections, we examined axon branching in the tdTomato-labeled layer 6b projections from the entire cortical mantle and with the cre-dependent AAV tracing from somatosensory cortex in addition to single-cell reconstructions. We also compared varicosity formation in the thalamus. The Cre-dependent AAV GFP-labeled 6b projections pass through TRN (Supplementary Fig. 3*B*) and VPL without side branches or synaptic boutons before branching at the medial edge of VPM nucleus and in Po. Similarly, for single cell tracing from BDA-labeled material, it is evident that the single layer 6b cell does not form side branches in TRN or VPL/VPM, but then extensively arbourises at the lateral edge of Po, with a few side branches projecting back into VPM (Fig. 5). Interestingly, the terminal arbourizations of 6b axons are far more spread within Po than single-cell labeled axons from L5b cells, which are markedly clustered on small tissue domains (Supplementary Fig. 6). The single-cell axon labeling shows that layer 6b terminal varicosities are small and mostly en passant, like those of axons from L6a cells (Figs 5 and 6). In contrast, most of the layer 5b terminals are much larger and are frequently located at the tip of a branch, as terminal varicosities (Fig. 6 and Supplementary Fig. 6). In contrast, virus injected L6a/6b (Ntsr1-Cre) brains (Supplementary Fig. 3*C*) and single cell BDA-labeled samples (Supplementary Fig. 7) show extensive branching in TRN and VPM, in agreement with the previous literature.

### Layer 6b Axons Form Small Synapses in Po

**Figure 7.**
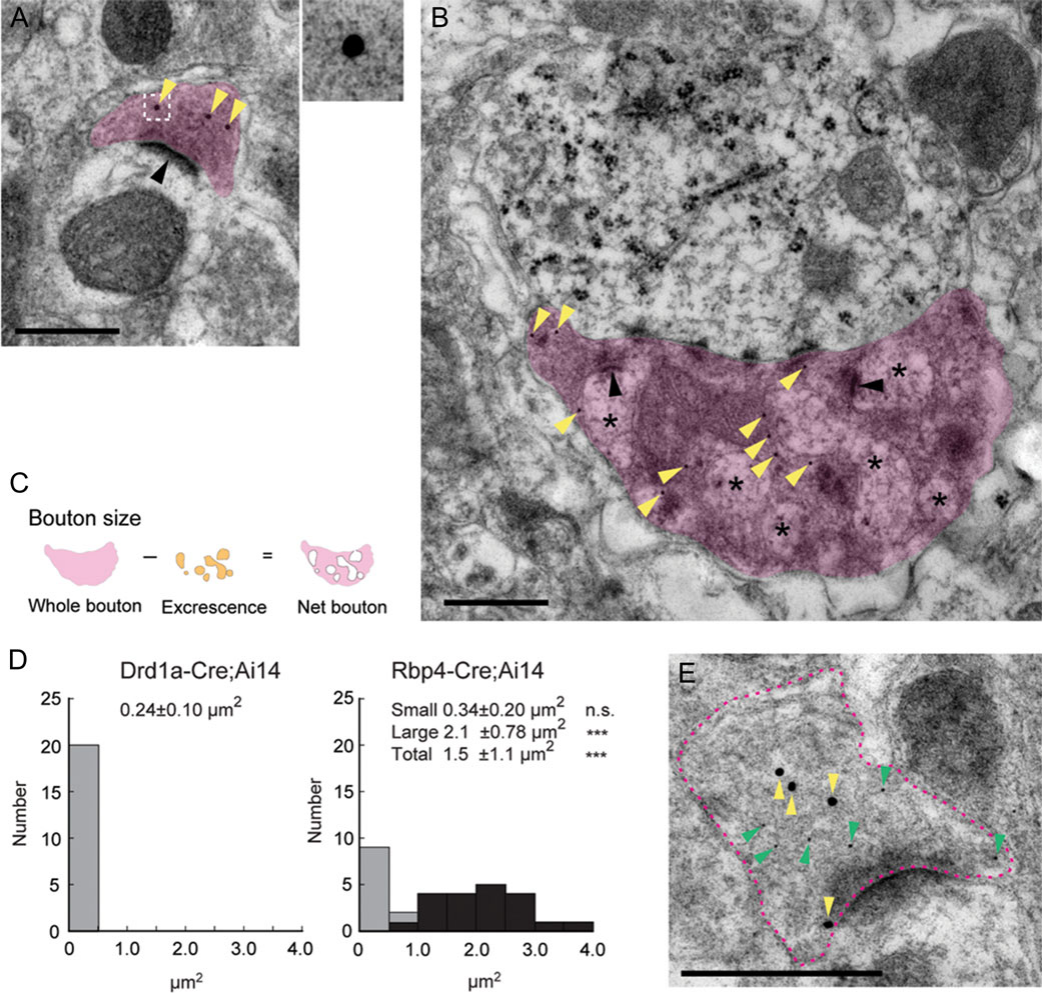
**Ultrastructural comparisons of Drd1a-Cre::tdTom^+^ and Rbp4-Cre::tdTom^+^ boutons in Po**. (*A,B*) Electron micrographs of Drd1a-Cre::tdTom^+^ (*A*) and Rbp4-Cre::tdTom^+^ (*B*) boutons labeled with postembedding immunogold against tdTomato. (*A*) A typical example of a Drd1a-Cre::tdTom^+^ bouton (magenta) that has a single asymmetric synapse (black arrowhead). Yellow arrowheads pointing to gold-particles labeling tdTomato. The boxed region in the left image is given at higher magnification on the top right to show a gold particle. (*B*) A typical example of a Rbp4-Cre::tdTom^+^ large bouton (magenta) that has multiple synapses (black arrowheads) and contains dendritic excrescences (asterisks) inside. (*C*,*D*) Quantitative analysis of the size of Drd1a-Cre::tdTom^+^ and Rbp4-Cre::tdTom^+^ boutons in Po. (*C*) For Rbp4-Cre::tdTom^+^ large boutons, the net area of the boutons excluding excrescences was measured. (*D*) Size distribution histogram of Drd1a-Cre::tdTom^+^ (left) and Rbp4-Cre;tdTom^+^ (right) boutons in single sections. The size of Drd1a-Cre::tdTom^+^ boutons is significantly smaller than that of the Rbp4-Cre::tdTom^+^ large boutons (right, black bars), but not different from that of the Rbp4-Cre::tdTom^+^ small boutons (right, gray bars). Dunn’s multiple comparison test. ***P < 0.001 (*E*) A Drd1a-Cre::tdTom^+^ axon that expresses VGluT1 in its bouton. Magenta dotted line outlines the bouton of a Drd1a-Cre::tdTom^+^ axon. Yellow and green arrowheads indicate 20 nm and 10 nm immunogold particles, which label tdTomato and VGluT1, respectively. Scale bars, 500 nm.

Presumed synaptic boutons in Po could be verified as VGluT1+ presynaptic terminals (Supplementary Fig. 3A, Fig. 7E), and mostly form as small side branches along an axon (Figs 5 and 6 and Supplementary Fig. 3A) in GFP virally-labeled material. Quantification of bouton size was performed on BDA-labeled material and on electron microscope images of immunogold-labeled tdTom^+^ synapses (see below). L6b, like L6a, cortico-thalamic axon varicosities in Po are “en passant” in BDA-labeled sections. L6b and 6a varicosities in Po have similar mean sizes (cross-sectional area 0.60 ± 0.01 μm^2^ and 0.58 ± 0.01 μm^2^, respectively; Mann–Whitney U test *P* = 0.930) and frequency of size distribution (K-S, *P* = 0.569). L5 cortico-thalamic axons, on the other hand, mostly form terminal axonal varicosities which are relatively scarce and significantly larger on average (2.87 ± 0.14 μm^2^, M–W U test, *P* < 0.001 for both comparisons), with sizes exceeding 10 μm^2^ on occasion, which also results in a significantly different frequency of size distribution (K–S, *P* < 0.001 for both comparisons). Thus, based on synapse size in the thalamus, layer 6b boutons are more similar to those of L6a, than the giant boutons derived from L5 cells. However, not all layer 5 synaptic terminals formed giant boutons; there were occasional small layer 5 terminals (with 0.2–1.5 μm^2^ cross-sectional area; Fig. 6*G*); whereas layer 6b terminals were never large.

We also performed ultrastructural characterization of Drd1a-Cre::tdTom^+^ axon projections in Po, to be able to better compare L5 and L6b derived terminals. Immunogold labeling of tdTom^+^ axons revealed that layer 6b axons form an asymmetric synapse in Po (Fig. 7*A*). We also observed Rbp4-cre::tdTom^+^ projections in Po and found that they have 2 different types of boutons. One is a bouton that contains a single asymmetric synapse, which is comparable to those of layer 6b. The other is a larger bouton that contains multiple synapses and is often invaded by dendritic excrescences (Fig. 7*B*), which has been reported in previous studies (Hoogland et al. 1991; Groh et al. 2008). We compared the size of Drd1a-Cre::tdTom^+^ boutons with that of Rbp4-Cre::tdTom^+^ boutons, which exclude the regions of dendritic excrescences (Fig. 7*C*). The size of Drd1a-Cre::tdTom^+^ presynaptic boutons in single sections are 0.24 ± 0.10 μm^2^ (*n* = 20 synapses from 2 brains, Fig. 7*D* left). The size of the Rbp4-Cre::tdTom^+^ small boutons are 0.34 ± 0.20 μm^2^ (*n* = 10 synapses from 2 brains, Fig. 7*D* right gray bars) and it is not significantly different from that of Drd1a-Cre::tdTom^+^ boutons. The size of the Rbp4-Cre::tdTom^+^ large boutons including and excluding dendritic excrescences are 2.8 ± 1.3 μm^2^ and 2.1 ± 0.78 μm^2^, respectively (*n* = 20 synapses from 2 brains, Fig. 7*D* right, black bars; Dunn’s test L6b vs. L5 small boutons, *P* > 0.05; L6b vs. L5 large boutons, *P* < 0.001; L6b vs. L5 all boutons, *P* < 0.001).). We also confirmed localization of VGluT1 in Drd1a-Cre::tdTom^+^ boutons by double immunolabelling of VGluT1 and tdTomato (Fig. 7*E*), confirming that these are excitatory glutamatergic synapses.

### Cortico-Cortical Projections from Layer 6b Neurons in Selected Cortical Regions

**Figure 8.**
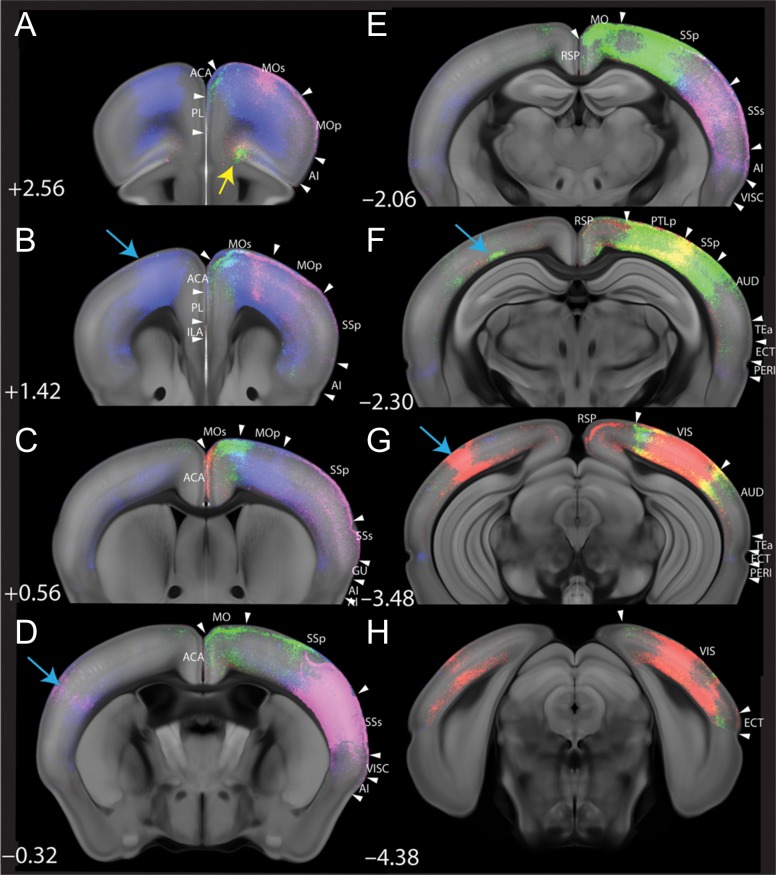
**Cre-dependent AAV2 tracing reveals areal specificity of layer 6b cortico-cortical projections in the adult Drd1a-Cre mouse**. Pseuo-colored AAV-eGFP infected areas in MO (blue), SS (magenta), SS/PTLp/VIS (green) and VIS (red) of Drd1a-Cre brains and their cortico-cortical projections; injections on right side of brain. The non-cortical projections are not included for clarity. All injected areas have contralateral projection in matched areas (blue arrows). Note the long-range projection in orbital cortex (yellow arrow in A) from SS/PTLp/VIS (green) and VIS (red). Ipsilaterally, cortical projection from different cortical domains shows topographically segregate targeting (C as an example). ACA, anterior cingulate area; AI, agranular insular area; AUD, auditory area; ECT, ectorhinal cortex; GU, gustatory area; ILA, infralimbic area; MOp, primary motor cortex; MOs, secondary motor cortex; PERI, perirhinal cortex; PL, prelimbic area; PTLp, posterior parietal association area; RSP, retrosplenial area; SSp, primary somatosensory area; SSs, secondary somatosensory area; TEa, temporal association area; VIS, visual area; VISC, visceral area.

To further characterize the connectivity of layer 6b cells, we also report the major intracortical projection patterns observed following anterograde viral tracing from 4 different cortical areas. For these 4 areas, distinct cortical projection patterns emerged (Fig. 8). We chose one representative sample from each injection area with high viral expression to capture long-range projections. The Drd1a-Cre expressing subset of layer 6b neurons from the motor area (blue in Fig. 8) strongly and bilaterally project to somatosensory region as well as lateral association cortices (temporal association areas (TEa), ectorhinal (ECT) and perirhinal (PERI) area). Somatosensory area (magenta in Fig. 8) projects ipsilaterally to motor cortex, other somatosensory and auditory regions, lateral association cortices (TEa, ECT) as well as across the corpus callosum to contralateral primary somatosensory cortex. Conversely, layer 6b neurons from the posterior SS/parietal (PTLp)/visual area (green in Fig. 8) target orbital cortex (yellow arrow in Fig. 8*A*), secondary motor cortex and lateral associational cortices, but largely avoid primary motor cortex. Projections derived from visual cortex (red in Fig. 8) also target orbital cortex (yellow arrow in Fig. 8*A*) as well as anterior cingulate cortex (ACA). All 4 areas showed contralateral projections to the area matching the injection site (light blue arrows in Fig. 8).

## Discussion

The thalamus receives input from 3 distinct cortical layers; inputs from 2 of these have been well characterized. Although this revealed heterogeneity in the L6 cortico-thalamic cell population in terms of their termination regions, this has not been systematically investigated. Here, we present evidence that the Tg(Drd1a-cre)FK164Gsat/Mmucd (Drd1a-Cre) line is useful to selectively study the projections of a subset of layer 6b neurons and distinguish them from layers 5 (Gong et al., 2007) and from models where both 6a and b are included (Olsen et al. 2012; Grant et al. 2016; Chevée et al. 2018). Since Drd1a-Cre labels a distinct subset of L6b, we do not know how representative these results are of all layer 6b projection neurons, nevertheless a similar projection pattern was confirmed with BDA single-cell tracing that is not dependent on specific Cre expression.

In this study, we examined the projections of Drd1a-Cre^+^ layer 6b cells in the adult brain with special attention to target nuclei in the thalamus. We observed that layer 6b neurons labeled in the Drd1a-Cre mouse are different from both layer 5 and layer 6 neurons labeled with Rbp4-Cre or Ntsr1-Cre; they selectively innervate higher order thalamic nuclei, do not branch in TRN and exclusively form small boutons with the hallmarks of glutamatergic synapses (VGluT1+ and asymmetric). We confirmed our results obtained with Cre-dependent viral tracing with single cell reconstructions of BDA-labeled layer 6b cortical neurons, labeling of which is not dependent on selective Cre expression. We also examined the terminals of the layer 6b projections labeled with different methods and compared these to the terminals formed by layer 5 cortico-thalamic projections at the ultrastructural level. Our study demonstrates that L6b projections to the thalamus are distinct from both L5 and L6a projections and represent a third type of cortico-thalamic projection.

### Characterization of Layer 6b Intracortical and Extracortical Projections

Crossing the Drd1a-Cre transgenic mouse to the Ai14 strain containing STOP-floxed tdTomato in the R26R locus, resulted in labeling of the entire Cre-expressing cell population, whereas injecting Cre-dependent GFP-expressing AAV2 into motor, somatosensory or visual cortex revealed layer 6b neuronal projections from the site of the injection.

For cortico-cortical projections, the Drd1a-Cre^+^ layer 6b neurons innervate distant cortical targets on the ipsilateral side in a topographically ordered fashion. Additionally, Cre-dependent viral labeling of layer 6b neurons reveals some axons crossing the corpus callosum, projecting to the equivalent areas contralaterally.

Similar to the cortico-cortical projections, layer 6b axons derived from visual and somatosensory cortex also followed distinct regional trajectories to subcortical targets. For all injection sites, the majority of the thalamic projection was ipsilateral to the injection site. Similar to layer 5 axons (Deschênes et al. 1994; Groh et al. 2014), layer 6b fibers from visual cortex densely innervate LP, and SS derived axons innervate Po. SS derived axons target more ventral thalamic nuclei than VIS derived axons, and the projection from mixed SS and visual areas also targets thalamic nuclei in between the pure SS and VIS targets. The rostral SS injections result in more ventral Po innervation compared to the mixed caudal SS/rostral VIS injection, suggesting even more refined topography within individual nuclei. MO-derived axons target more medial thalamic nuclei than SS or VIS derived axons, and form denser and more widespread bilateral projections.

### Thalamic Projections of Layer 6b Are Different from Both Layer 6a and Layer 5

Examining the fiber pattern in the Drd1a-Cre;Ai14 mouse revealed that layer 6b neurons target the thalamus with a clear separation between first and higher order thalamic nuclei. Generally, L6b projections densely innervate midline and higher order thalamic nuclei, but form few side branches or synapses in first-order thalamic nuclei. This is in clear contrast to the single-cell tracing reported by Bourassa and colleagues, where cells in “the lower half of L6” of somatosensory cortex form collaterals in VPM (Bourassa et al. 1995). In our material, some first-order nuclei appear to contain hardly any tdTom^+^ fibers throughout, yet are strongly labeled by viral injections in border regions adjacent to higher order thalamic nuclei, for example, medial edge of VPM. Most higher order thalamic nuclei on the other hand, are densely innervated by tdTom^+^ fibers, and AAV2 labeled GFP^+^ fibers from appropriate cortical regions form very dense patches of innervation covering contiguous smaller regions of such higher order nuclei. The Rbp4-Cre^+^ layer 5 population targets higher order thalamic nuclei (Grant et al., 2016), but more detailed region-specific AAV2 tracing is required to explore possible regional differences. Despite the similarities in L5 and L6b projection distributions, we did not find evidence of Cre-expressing cells in L5 in the cortical regions examined, including motor and somatosensory cortex.

A recent study of Viswanathan et al. (2016) suggested that layer 6b projections have association to higher order thalamic nuclei based on the distribution of Complexin 3 (Cplx3) immunoreactive terminals in the thalamus. Indeed Cplx3 is strongly expressed in layer 6b cortical neurons, but it is also expressed in some layer 5 populations (Hoerder-Suabedissen et al., 2009, 2013; Belgard et al., 2011). Therefore, while the much higher numbers of Cplx3 layer 6b cells suggest that Cplx3 labeled projections in thalamus are likely to originate from layer 6b, a contribution of layer 5 to this terminal immunoreactivity cannot be ruled out completely. Our use here of a selective layer 6b transgenic line resolves this potential confound.

The tdTom^+^ fibers in thalamus derive from the entire cortical mantle, and do not permit characterization of the temporal innervation patterns of thalamus from different cortical regions. Given the onset of Cre-recombinase expression is after P3 for most cortical cells, we did not attempt to identify temporal patterns of thalamic innervation by different cortical regions, as the adult-like pattern is established by P21 at the latest. It would be very interesting to characterize the early ingrowth of layer 6b fibers into the thalamus, but the Drd1a-Cre strain is not the best tool to do so. Although we can demonstrate that at least some layer 6b fibers reach Po thalamus by P6, it remains unclear whether the projection itself is very sparse at this age, or whether the cells are sparse due to a relatively late switch-on of Cre expression in their development. Therefore, the majority of this paper and the following discussion is focused on the adult projection pattern and putative adult functions of this projection.

The cortico-thalamic afferent contribution to innervation of Po has been previously studied in some detail in the rat (Veinante et al. 2000; Alloway et al. 2003; Killackey and Sherman 2003). Injections of retrograde tracer had revealed abundant backlabeled cells in layer 6b, across the lateral expanse of S1 cortex (Killackey and Sherman 2003; Chevée et al. 2018). The same authors noted a near absence of backlabeled cells in layer 6b when the injection was into VPM. An earlier study utilizing single cell reconstructions of neurons in S1 also identified 2 cells just above the white matter with Pom axonal arbors without collaterals in VPM or TRN (Bourassa et al. 1995; reviewed in Deschênes et al. 1998), although the majority of the “deep layer 6” cells were reported to form collaterals both in VPM and TRN. In addition, injections of retrograde tracers into LP backlabel layer 6b neurons in visual cortex (Roth et al. 2016). Based on our results of labeling a subset of layer 6b neurons transgenically as well as from BDA-labeled single cell reconstructions from S1, we confirm that a subpopulation of layer 6b neurons does not innervate TRN, may form side branches in VPL, but only rarely forms side branches in VPM, before densely arbourizing in Po, a projection pattern previously suggested for a different transgenic strain, too (Shima et al. 2016). Layer 6b projections originating in VIS project to LP (and LD).

The distribution of cortical cells innervating various thalamic nuclei has been studied in considerable detail in primates, especially in the context of prefrontal cortex. Direct comparisons to the mouse is often difficult, as it is frequently not clear whether “deep layer 6” neurons would be the equivalent of mouse layer 6b cells, and the white/gray matter boundary is not always evident in the photomicrographs of older publications. However, there are a few exceptions to this rule, where cells are clearly referred to as “white matter neurons” or depicted as such. For example, sparse “white matter” neurons in cingulate, prefrontal cortex and supplementary motor area were retrogradely labeled following tracer injections into MD or ventral anterior (VA)/ventral lateral (VL) nucleus in adult rhesus monkeys (Giguere and Goldman‐Rakic 1988; Xiao et al. 2009). Whether corticofugal projections from “white matter” neurons in primates avoid first-order thalamic nuclei is less clear, as there are no comprehensive studies of white matter neuron projections to our knowledge. However, Usrey and Fitzpatrick (1996) report that retrograde tracing from pulvinar selectively labeled “deep layer 6” neurons, in contrast to injections into LGd, which labeled neurons across the depth of layer 6 (Usrey and Fitzpatrick 1996). Thus, while there is some evidence for similarity of corticofugal layer 6b projections in rodents and white matter neuron projections in primates for a few selected regions, a comprehensive comparison would be required to draw any firm conclusions.

### Contralateral Projections from Layer 6b

The existence of contralateral projections from subplate/layer 6b neurons has been controversial, and may be species dependent. Contralaterally projecting subplate cells exist in ferrets (Antonini and Shatz 1990). For embryonic rodents, rare contralateral projections from subplate were reported (Ozaki and Wahlsten 1998), but there is disagreement about their existence in the postnatal period. Both absence (Robertson 2000) and presence (Hoerder-Suabedissen and Molnár 2012) of contralateral projections from postnatal subplate cells in rodents have been reported. Additionally, in the Golli-τ-eGFP (GTE) mouse in which pioneer neurons transgenically express eGFP, labeled fibers are present in the corpus callosum and the anterior commissure (Jacobs et al. 2007; Grant et al. 2016), but it is not clear whether the GFP-positive fibers derive from layer 6b or the layer 6a neurons which are known to form projections to contralateral cortex (Koester and O’Leary 1994). Here we report that the method of axonal labeling may influence whether callosal projections are detectable in adult brains. Using the same Drd1a-Cre transgenic strain, callosal fibers are not visible with the red and relatively weak genetic tracer Stop-floxed-Ai14 (which adequately reveals abundant subcortical projections), but are visible in 2 of 4 brains (both of which had large injections) when using Cre-dependent AAV-GFP viral tracing from SS in adult brains. Additionally, contralateral axonal branches are clearly visible in some brains with viral injections into VIS, but the point of midline crossing is not always identifiable. MO injections give rise to contralateral axonal arbors in motor regions, with clear callosal trajectories.

### Size of Layer 6b Thalamic Terminal Varicosities

Layer 5 terminals are frequently described as ‘giant’ terminal varicosities of considerable size (3–5 μm in Po (Bourassa et al. 1995; Liao et al. 2010)). While this size range is in agreement with our EM measurements and our BDA-labeled samples, not all layer 5 terminals are this large, there are layer 5 terminals with much smaller diameter as well. In contrast to layer 5, we found that all L6b terminal varicosities are considerably smaller, and more similar in size to L6a varicosities (<1 μm^2^ (Bourassa et al. 1995; Zhang and Deschênes 1997; Deschênes et al. 1998)).

There are major physiological differences between the layer 5 and layer 6a terminals in the thalamus. Layer 5 thalamic projections target closer to the cell body, act via ionotropic glutamate receptors, and demonstrate paired pulse inhibition after repeated stimulation. Layer 6a projections target more distally, act via metabotropic as well as ionotropic glutamate receptors, and show paired pulse facilitation on repeated stimulation (Sherman and Guillery 1996). Such characterizations are yet to be performed on the layer 6b terminals.

### Function of Layer 6b Thalamic Projections

What is the function of layer 6b thalamic projections to the higher order thalamic nuclei? Layer 6a projections control the thalamus with dynamic synapses, depending upon their firing frequency (Crandall et al. 2015). Layer 6a projections may stimulate the thalamic projection neurons directly (Przybyszewski et al. 2000) and inhibit them through stimulating the TRN with their collaterals. The net effect can be excitatory or inhibitory (Denman and Contreras 2015). Depending on the balance of these influences, they modulate the sensory gating (Crandall et al. 2015), and a sharpening of the orientation tuning of LGd cells has been reported (Andolina et al. 2007). Layer 6b projections from Drd1a-cre^+^ neurons, on the other hand, cannot elicit inhibition in TRN since they do not have side branches or synapses in TRN. Like layer 6b, layer 5 mostly provides input to higher order thalamic nuclei. The layer 5 input is via axon collaterals of subcortical projections and they are mostly powerful and large, feed-forward “driver” inputs for relay to other cortical areas (Sherman and Guillery 1996; Rouiller and Welker 2000; Reichova and Sherman 2004; Groh et al. 2008). Our work revealed that some terminals from Rbp4-cre^+^ layer 5 projections were small. Their origin and function require more detailed investigations. However, our analysis also revealed that layer 6b terminals from Drd1a-cre^+^ neurons in Po are always small in contrast to layer 5 projections. Their participation in transthalamic cortical loops shall have to be further studied. There is evidence from rabbit and cat that “deep layer 6” neurons, that may include the equivalent of the mouse layer 6b cells, have particularly slow axonal conduction velocities (Tsumoto and Suda 1980; Swadlow 1994; Stoelzel et al. 2017). Taken together, this suggests that Drd1a-cre^+^ layer 6b projections are more likely to be involved in modulating rather than driving the higher order thalamic nuclei. The layer 6b projections could act as stimulatory counterparts of zona incerta projections that also selectively target the higher order thalamic nuclei (Barthó et al. 2002; Mitrofanis 2005), or projections to the higher order thalamic nuclei may be important for selectively recruiting an expanded network of cortical areas and/or changing their functional state (Schmitt et al. 2017) in order to cope with conflicting/demanding inputs. Besides, the observation that both layer 6b neurons and higher order thalamic nuclei neurons are selectively sensitive to orexin suggests that such recruitment might fluctuate depending on the level of arousal. The novel selective projection to higher order thalamic nuclei, combined with the potentially arousal dependent activity state of layer 6b neurons highlights the necessity of further physiological analysis of this enigmatic cell group in awake, behaving animals.

## Supplementary Material

Supplementary Data
