# Supplementary material for: Subset of Cortical Layer 6b Neurons Selectively Innervates Higher Order Thalamic Nuclei in Mice
Source: Cereb Cortex. 2018 Feb 22;28(5):1882–97. doi: 10.1093/cercor/bhy036 (PMC6018949; doi:10.1093/cercor/bhy036)
Supplement: Supplementary Data [file bhy036suppl_1.zip › SupplTextandFigurelegends.docx]

**SUPPLEMENTARY MATERIALS**

**Supplementary Methods**

Retrograde labelling with aminostyryl dye in fixed tissue

DiA crystals (4-(4-(dihexadecyl-amino)styryl)-N-methylpyridinium iodide; D3883; Molecular Probes) were placed in Po of P6 (N=1) or P8 (n=2) Drd1a-Cre;Ai14 brains, following removal of the hindbrain and caudal pole of the forebrain. Brains were kept at room temperature in 0.1MPBS + 0.1% sodium azide for 6-7 weeks for the dye to diffuse. Brains were cut to 70μm coronal, sections counterstained with DAPI and mounted in 0.1M PBS before being coverslipped. Imaging (and cell counting) took place within 12hrs of mounting the sections.

**Supplementary Table legends**

**Supplementary Table 1. Summary of target region and number of animals injected for each strain.** Summary table indicating the stereotaxic coordinates used to inject different mice with Cre-dependent AAV. Also indicated are the different mouse strains, the quantity of virus used and the number of animals with each type of injection included in this study.

**Supplementary Table 2. Summary of antibodies used in this study**. List of antibodies used, including concentration, species they were raised in and the product details. We highlight unusual experimental conditions or additional steps in the protocol in the “additional comments” fields.

**Supplementary Table 3. Summary of Cre-expression outside the central nervous system in adult Drd1a-Cre;Ai14 mouse.** All identifiable structures with positive findings are included in the table, and some description is given of the type of labelling observed. Some individual organs that were completely unlabelled are mentioned for completeness. Faint red glow is indicated as “+”, bright labelling of a subset of cells within the organ or intermediate uniform glow is indicated as “++” and dense and bright labelling within an organ is given as “+++”. Data documented in two adult Drd1a-Cre::tdTom+ males.

**Supplementary Figure legends**

**Supplementary Figure 1. Non-cortical Cre-expression in adult brains.** Cre-expression in the brain was documented in adult Drd1a-Cre;Ai14+ mice at 5 months of age. (A, A’) in adult brains, a few scattered tdTom+ cells are found in the striatum (arrows). (B, B’) Similarly, sparse tdTom+ pyramidal cells are located throughout the hippocampus (arrows), occasionally a few Cre+ cells are found in the ventral lateral geniculate nucleus (vLG; arrowheads) and dense cells are located in the medial pretectal area (asterisk). (C,C’) More caudally, tdTom+ cells are abundant in the hippocampal and subicular pyramidal layer (arrows). (D, D’) In the cerebellum, tdTom+ cells are extremely abundant in the dorsal cochlear nucleus (asterisk), present in the flocculus and paraflocculus (arrows) and sparsely in the granular layer of crus II (arrowheads) and occasionally present in other cerebellar lobules. Scale bar = 1mm.

**Supplementary Figure 2. tdTom+ layer 6b cells extend axons towards Po thalamus by P6.** A crystal of carbocyanine dye DiA was placed into Po of an intact, fixed Drd1a-Cre;tdTom brain at P6. (A) Crystal placement site is indicated by the asterisk (DiA signal is shown in green). (B) The arrow points to the location of the backlabelled cell shown in (C). (C) A DiA-backlabelled cell in the subplate that is also tdTom+ (arrow). Note the nearby tdTom+ cells that are not backlabelled, as well as the DiA+tdTom- cells higher up in the cortex (arrowhead). Scale bars = 500μm (A,B) and 50μm (C).

**Supplementary Figure 3.** **Layer 6b cells in SS form small synapses in Po but not TRN**. To compare projections from layer 6b and layer 6a we injected 200nl AAV2-CAG-Flex-ArchT-GFP virus into SS cortex of Drd1a-Cre;Ai14 or Ntsr1-Cre;Ai14. Laser scanning confocal images (tiled) and orthogonal view projections of image stacks. (A) GFP+ axons from layer 6b (Drd1a-Cre) form VGluT1+ synapses in Po thalamus. The arrowheads in the inset and main panel point to the same synaptic terminal. (B) GFP+ L6b (Drd1a-Cre) axons were not observed to branch in TRN (data not shown), nor is there evidence of side-branch formation on the tdTom+ axons. Dense branching is visible at the lateral edge of VPL. No overlap between VGluT1+ synapses and tdTom+ axons was observed in TRN (inset). (C) In comparison, Ntsr1-Cre::tdTom+ axons branch profusely in TRN, and there is overlap between VGluT1+ synapses and tdTom+ axon side-branches. The arrowheads in the inset and main panel point to the same synaptic terminal. For all images, dorsal is top, lateral is left. Scale bars = 10μm (A) or 40μm (B, C).

**Supplementary Figure 4. Summary of the Cre-dependent AAV2 tracing results revealing areal specificity of layer 6b cortico-cortical and subcortical projections in the adult Drd1a-Cre mouse**. Schematic circles on the cortex represent the injections on the right side of brain to the four areas studied; MO (blue), SS (magenta), SS/PTLp/VIS (green) and VIS (red). Cre-dependent viral injections to these areas of Drd1a-Cre brains revealed the projections of a subpopulation of layer 6b neurons with their intracortical and extracortical projections. Ipsilaterally, cortical projection from different cortical domains show topographically segregated targeting. Note the long-range projection to orbital cortex (ORB) and anterior cingulate cortex (ACA) from SS/PTLp/VIS (green) and VIS (red). There are projections to the lateral association cortex from the primary visual cortex. Additionally, all injected areas have projections to equivalent areas contralaterally. MO and VIS also project to orbitofrontal cortex contralaterally, and SS/VIS and VIS project to anterior cingulate cortex in the opposite hemisphere. MO has additional long-range projections to lateral association areas on the contralateral side. Furthermore, all cortical areas had ipsilateral projections to thalamus, with a smaller contralateral component, mostly in midline thalamic nuclei (not summarised in detail here). The Drd1a-cre+ layer 6b neurons of the primary visual cortex also projection to the superior colliculus (SC), and L6b cells from MO project to substantia nigra (SN), pretectum (PRT) and superior colliculus. Both VIS and MO cortex project to caudate putamen (CP) bilaterally. The schematic outline drawing is adapted from the original drawing by Larry Swanson (http://larrywswanson.com/?page_id=1415).

**Supplementary Figure 5. Comparison of the labelling patterns obtained from SS injections of Cre dependent AAV into layer 6b specific Cre driver Drd1-Cre with the patterns obtained in Layer 5 (Rbp4-Cre) and Layer 6a (Ntsr1-Cre) mice.** (A) AAV infected areas of L5 specific Rbp4-Cre (left; data from Allen Institute), L6a specific Ntsr1-Cre (middle; data from Allen Institute), and L6b specific Drd1-Cre (right) targeting the somatosensory cortex. (B) Thalamic projection summary. Projection data from different Cre drivers are pseudo-coloured to distinguish different projections. Note that both Rbp4- and Ntsr1-Cre target both higher order and primary thalamic nuclei while Drd1-Cre most selectively innervates the higher order thalamus. Numbers at the bottom represent anterior-posterior bregma coordinates. Abbreviations: CL: central lateral nucleus; LD: lateral dorsal nucleus; LP: lateral posterior nucleus; PCN: paracentral nucleus; PF: parafascicular nucleus; PO: posterior nucleus; RE: nucleus reuniens; RH: rhomboid nucleus; SPF: subparafascicular nucleus; RT: (pre-)thalamic reticular nucleus; SMT: submedial nucleus; VAL: ventral anterior lateral complex; VM: ventral medial nucleus; VPL: ventral posterior lateral nucleus; VPM: ventral posterior medial nucleus.

**Supplementary Figure 6.** **Single-cell BDA-labeling of a layer 5b projection neuron.**

(A) Image taken from a coronal section of S1BF containing the microiontophoresis site in layer (“L”) 5b. Cytochrome oxidase counterstain to delinate barrels in L4 of S1BF. The deposit center is visible as an amorphous black precipitate. Adjacent to it, the soma and dendritic arbour of a labeled L5b cell (arrowhead) is visible.

(B) Camera-lucida reconstruction of the somatodendritic arbour (blue) and axon (red) of the above L5b cell. Borders between cortical layers (L4-L6b) in cortex are delineated. Abbreviations: AP: Distance to Bregma in the anteroposterior axis, in mm. CPu: Caudate-putamen; Ic: Internal capsule; TRN: Reticular (pre-)thalamic nucleus; Po: Posterior thalamic nucleus; VPL: Ventral posterolateral thalamic nucleus; VPM: Ventral posteromedial thalamic nucleus; WM: White matter.

(C) Photomontage showing the axon as it traverses TRN (inset in B). Arrowheads indicate the lateral and medial edges of TRN. Note the absence of side branches.

(D) Details of the labelled terminal axonal arbourisation into Po. Scale bars: A= 150µm; B =500µm; C = 50µm; D=20µm.

**Supplementary Figure 7.** **Single-cell BDA-labeling of a layer 6a neuron.**

(A) A coronal section of S1BF containing the microiontophoresis site in layer (“L”) 6a. Cytochrome oxidase counterstain to delineate barrels in L4 of S1BF. In this section several somata and dendritic arbours of BDA filled L6 cells are visible.

(B) Camera-lucida reconstruction of the somatodendritic arbour (blue) and axon (red) of a single L6a cell (arrowhead in A). Borders between cortical layers (L4-L6b) in cortex are delineated. Abbreviations: AP: Distance to Bregma in the anteroposterior axis, in mm. CPu: Caudate-putamen; Ic: Internal capsule; TRN: Reticular prethalamic nucleus; Po: Posterior thalamic nucleus; VPL: Ventral posterolateral thalamic nucleus; VPM: Ventral posteromedial thalamic nucleus; WM: White matter.

(C) Details of the labelled terminal axonal arbourisation in TRN (inset in B marked by a cross), and (D) details of the labelled terminal axonal arbourisation in VPM (inset in B marked by an asterisk). Scale bars: A= 150µm; B =500µm; C = 25µm; D=50µm.
