# Supplementary material for: Subset of Cortical Layer 6b Neurons Selectively Innervates Higher Order Thalamic Nuclei in Mice
Source: Cereb Cortex. 2018 Feb 22;28(5):1882–97. doi: 10.1093/cercor/bhy036 (PMC6018949; doi:10.1093/cercor/bhy036)
Supplement: Supplementary Data [file bhy036suppl_1.zip › Suppl_Table2antibodies_new.docx]

**Supplementary Table 2. Summary of antibodies used in this study.**

| Target | Concentration | Host species | Manufacturer and product code | Secondary antibody | Additional comments |
| --- | --- | --- | --- | --- | --- |
| Cplx3 | 1:1000 | Rabbit | Synaptic Systems 122002 | Donkey α-rabbit AF488 |  |
| Cre | 1:1000 | Mouse | Sigma C7988 | Donkey α-mouse AF488 |  |
| CTGF | 1:1000 | Goat | Santa Cruz SC-14939 | Donkey α-goat AF488 | 1% Triton-X100 in block |
| Neuroserpin | 1:2000 | Goat | Abcam Ab32901 | Donkey α-goat AF488 |  |
| RFP | 1:500 | Rabbit | MBL International PM005 | Donkey anti-rabbit AF568  Goat-α-rabbit-Gold(20nm) | EM use only |
| VGluT1 | 1:10000 (fluorescence) or 1:500 (EM) | Guinea pig | Chemicon AB5905 | Donkey α-gp biotin or  goat-α-gp-Gold (10nm) | Followed by streptavidin-cy5 for fluorescence |

List of antibodies used, including concentration, species they were raised in and the product details. We highlight unusual experimental conditions or additional steps in the protocol in the “additional comments” fields.
