# Supplementary material for: Subset of Cortical Layer 6b Neurons Selectively Innervates Higher Order Thalamic Nuclei in Mice
Source: Cereb Cortex. 2018 Feb 22;28(5):1882–97. doi: 10.1093/cercor/bhy036 (PMC6018949; doi:10.1093/cercor/bhy036)
Supplement: Supplementary Data [file bhy036suppl_1.zip › Suppl_Table3_NonBrainexpression.docx]

| **Organ** | **Expression strength** | **Expression pattern restrictions** |
| --- | --- | --- |
| Back muscles | ++ | But not in intercostals |
| Big intestine | ++ | Individual cells |
| Bones | No | Not observed in exposed ribcage |
| Cartilage | +++ | Base of ear, tip of sternum (and see comment on tail) |
| Fat | + |  |
| Genitalia (male, external) | +++ |  |
| Genitalia (male, internal) | +++ |  |
| Heart | +++ | In two small patches on atrium and tip of ventricle |
| Kidney | ++ | Individual bright cells |
| Limbs | ++ | In lower hind limb but not heel |
| Liver | No |  |
| Lung | +++ | In small patches, but most of lung is unlabelled |
| Muscle | ++ | In inside back muscles, but not in intercostals |
| Paw-pads/digit pads | +++ | Not in the bones of the foot |
| Skin (Pinna of ear, top of foot) | No |  |
| Spleen | No |  |
| Tail | +++ | Alternating stripes suggesting either vertebrae or disks, but not both on the distal end, very bright on the ventral side at the junction with the body |
| Teeth/Mandible | +++ | Inside the mouth, at the junction of the incisors and lower jaw |

**Supplementary Table 3. Summary of Cre-expression outside the central nervous system in adult Drd1a-Cre;Ai14 mouse.** All identifiable structures with positive findings are included in the table, and some description is given of the type of labelling observed. Some individual organs that were completely unlabelled are mentioned for completeness. Faint red glow is indicated as “+”, bright labelling of a subset of cells within the organ or intermediate uniform glow is indicated as “++” and dense and bright labelling within an organ is given as “+++”. Data documented in two adult Drd1a-Cre::tdTom+ males.
