# Supplementary material for: Subset of Cortical Layer 6b Neurons Selectively Innervates Higher Order Thalamic Nuclei in Mice
Source: Cereb Cortex. 2018 Feb 22;28(5):1882–97. doi: 10.1093/cercor/bhy036 (PMC6018949; doi:10.1093/cercor/bhy036)
Supplement: Supplementary Data [file bhy036suppl_1.zip › Suppl_Table1-injectedregions_new.docx]

**Supplementary Table 1. Summary of target region and number of animals injected for each strain.**

| Strain | Region | Bregma | Laterality | Depth | Volume | Number of animals |
| --- | --- | --- | --- | --- | --- | --- |
| Drd1a-Cre | MO | 1.35 rostral | 1.75 left | 1.0-1.1 | 200nl | 4 |
| Drd1a-Cre;Ai14 | SS | 1.5 caudal | 3.0 left | 1.4-1.6 | 200nl | 5 |
| Drd1a-Cre;Ai14 | SS/VIS | 3.55 caudal | 2.25 left | 0.8 | 200nl | 3 |
| Drd1a-Cre | VIS | 4.2 caudal | 2.5 left | 0.6 | 200nl | 3 |
| Ntsr1-Cre;Ai14 | SS | 1.5 caudal | 2.5 left | 1.05-1.15 | 200nl | 4 |
| Rbp4-Cre;Ai14 | SS | 1.5 caudal | 2.8-3.0 left | 0.8 | 200nl | 5 |

Summary table indicating the stereotaxic coordinates used to inject different mice with Cre-dependent AAV. Also indicated are the different mouse strains, the quantity of virus used and the number of animals with each type of injection included in this study.
