# Supplementary figures and images for: Subset of Cortical Layer 6b Neurons Selectively Innervates Higher Order Thalamic Nuclei in Mice
Source: Cereb Cortex. 2018 Feb 22;28(5):1882–97. doi: 10.1093/cercor/bhy036 (PMC6018949; doi:10.1093/cercor/bhy036)

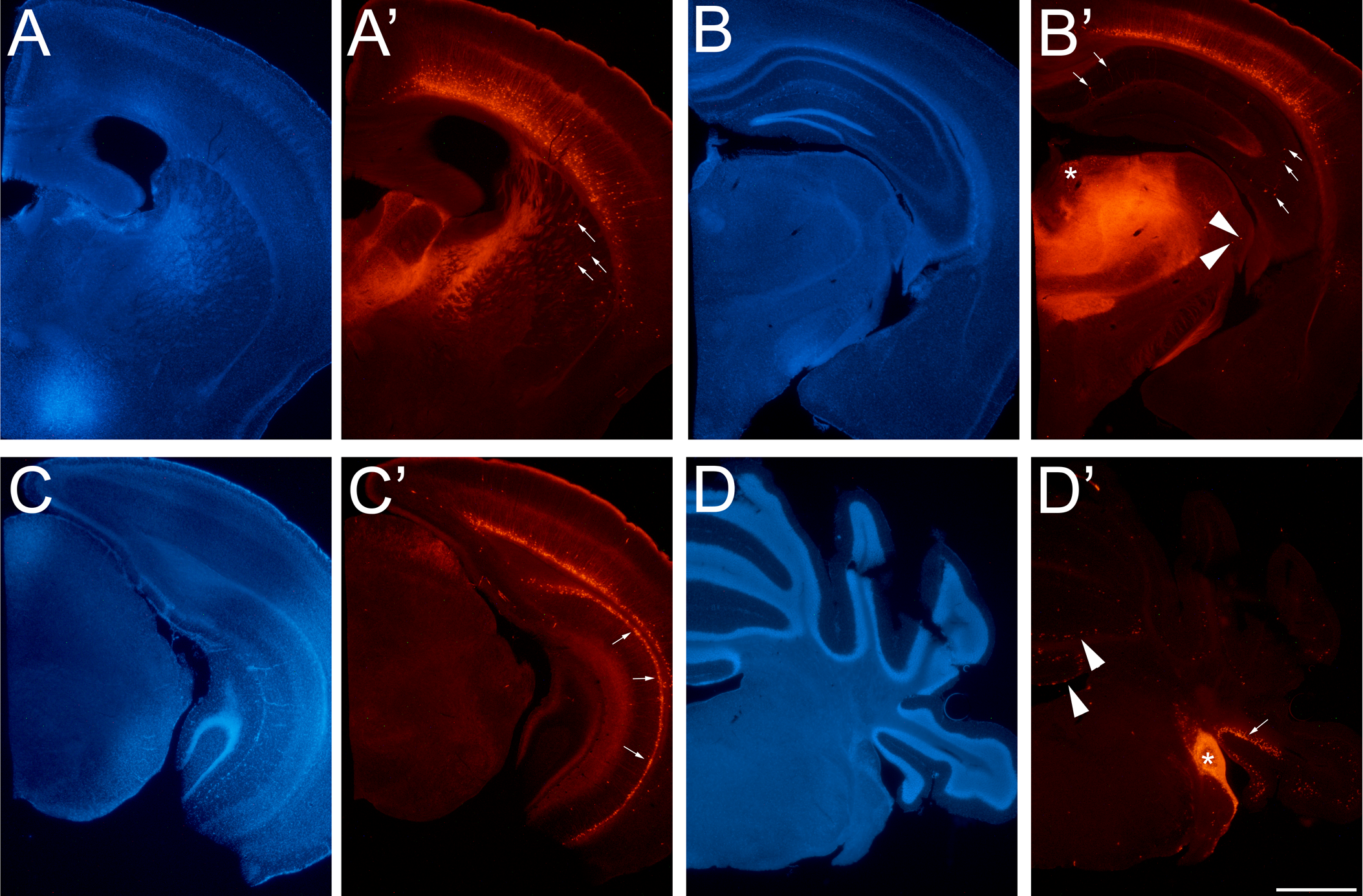

Supplement: Supplementary Data [file bhy036suppl_1.zip › Suppl_Figure1a_Non-corticalexpressioninadultbrain.tif]

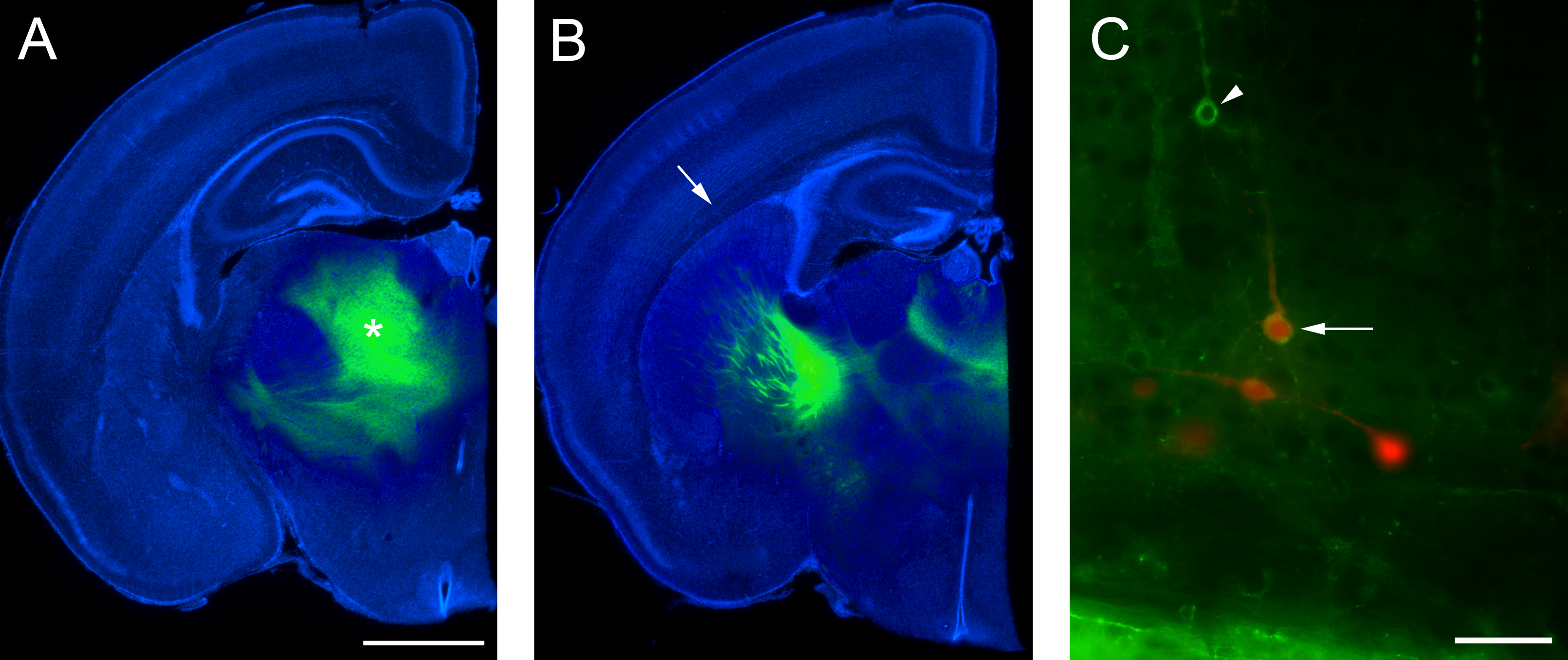

Supplement: Supplementary Data [file bhy036suppl_1.zip › Suppl_Figure2_Po-DiAlabelledcellinSPofS1.tif]

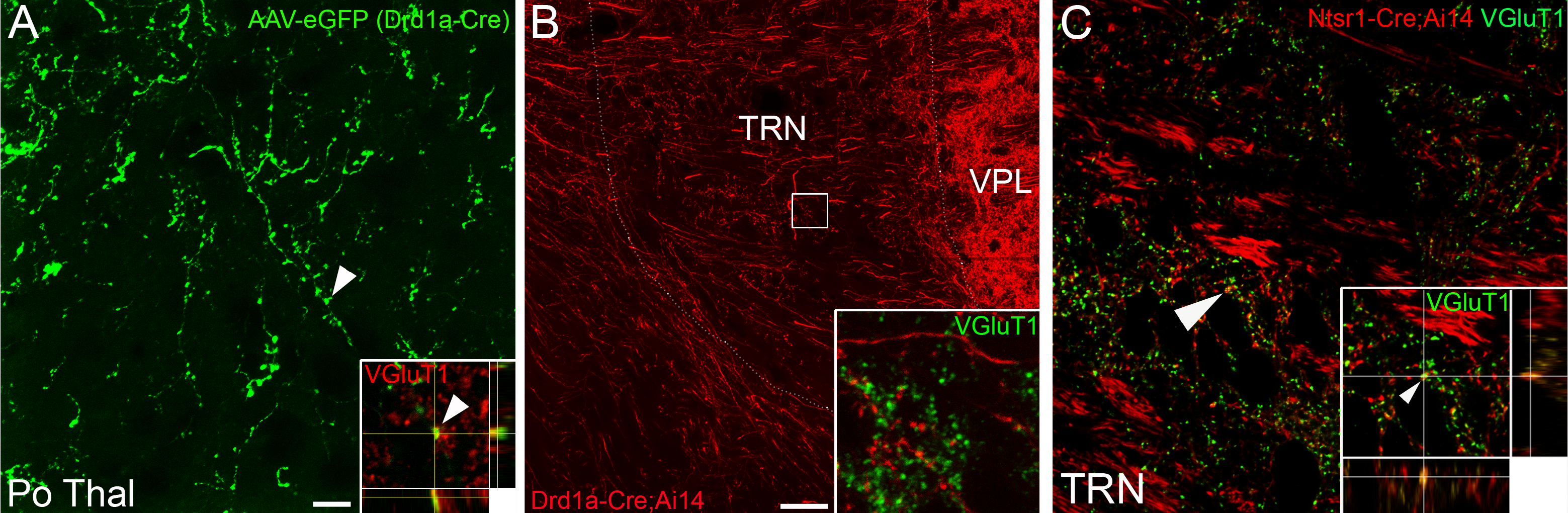

Supplement: Supplementary Data [file bhy036suppl_1.zip › Suppl_Figure3_new_VGluT1synapsesinPObutnotTRN.tif]

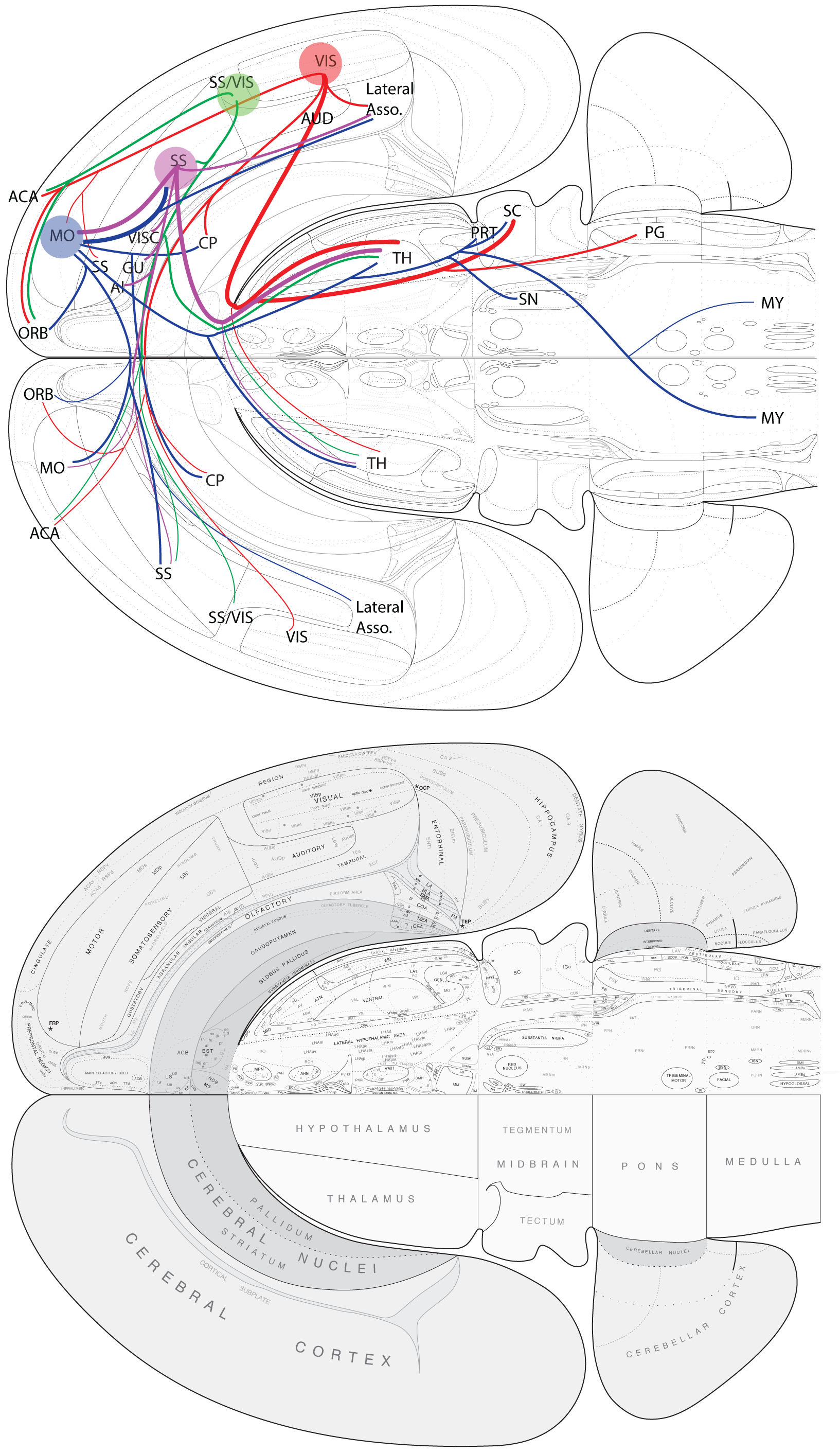

Supplement: Supplementary Data [file bhy036suppl_1.zip › Suppl_Figure4_projection_summary_WholeFlatmap.tif]

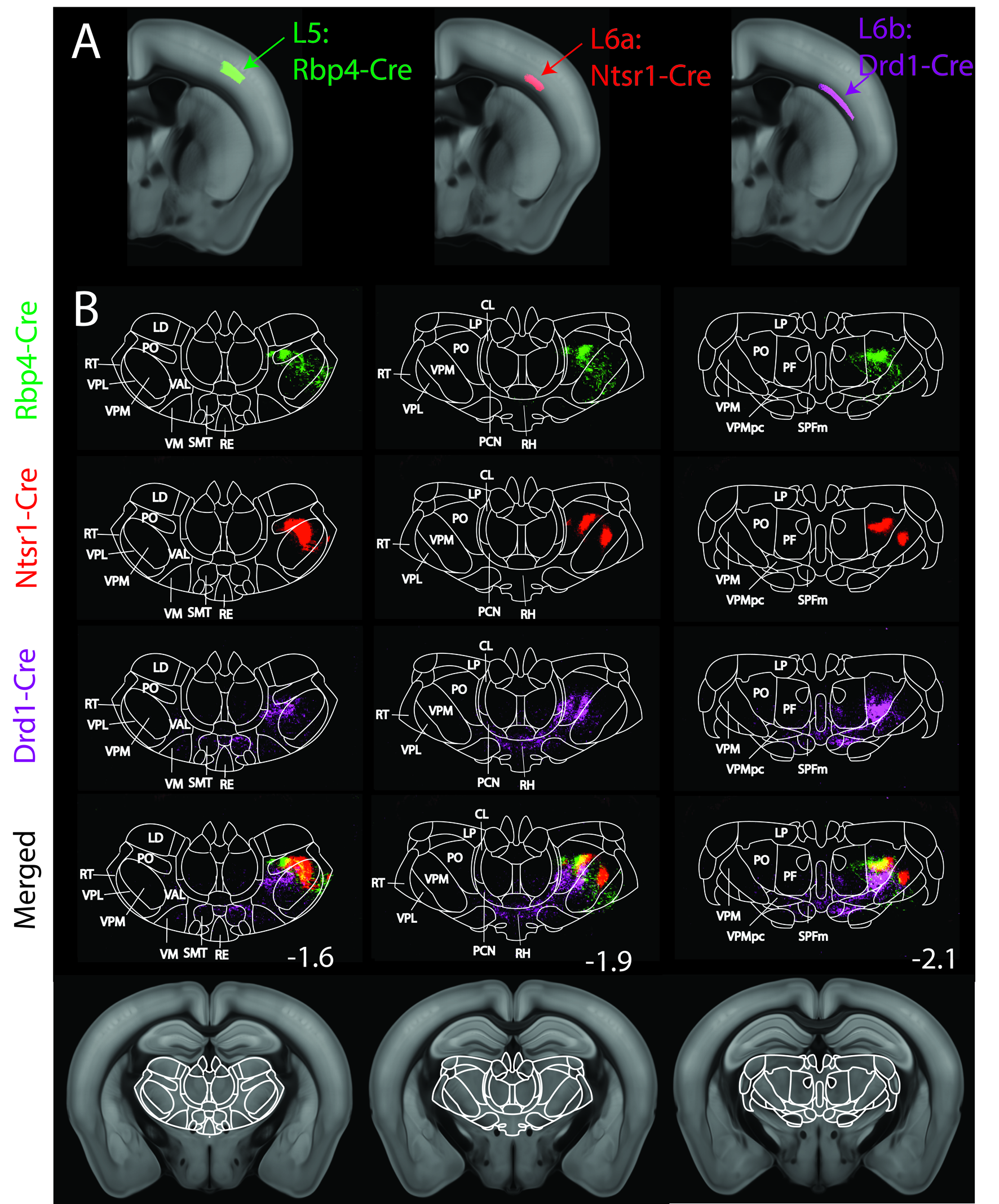

Supplement: Supplementary Data [file bhy036suppl_1.zip › Suppl_Figure5a_DifferentCre_comparison.tif]

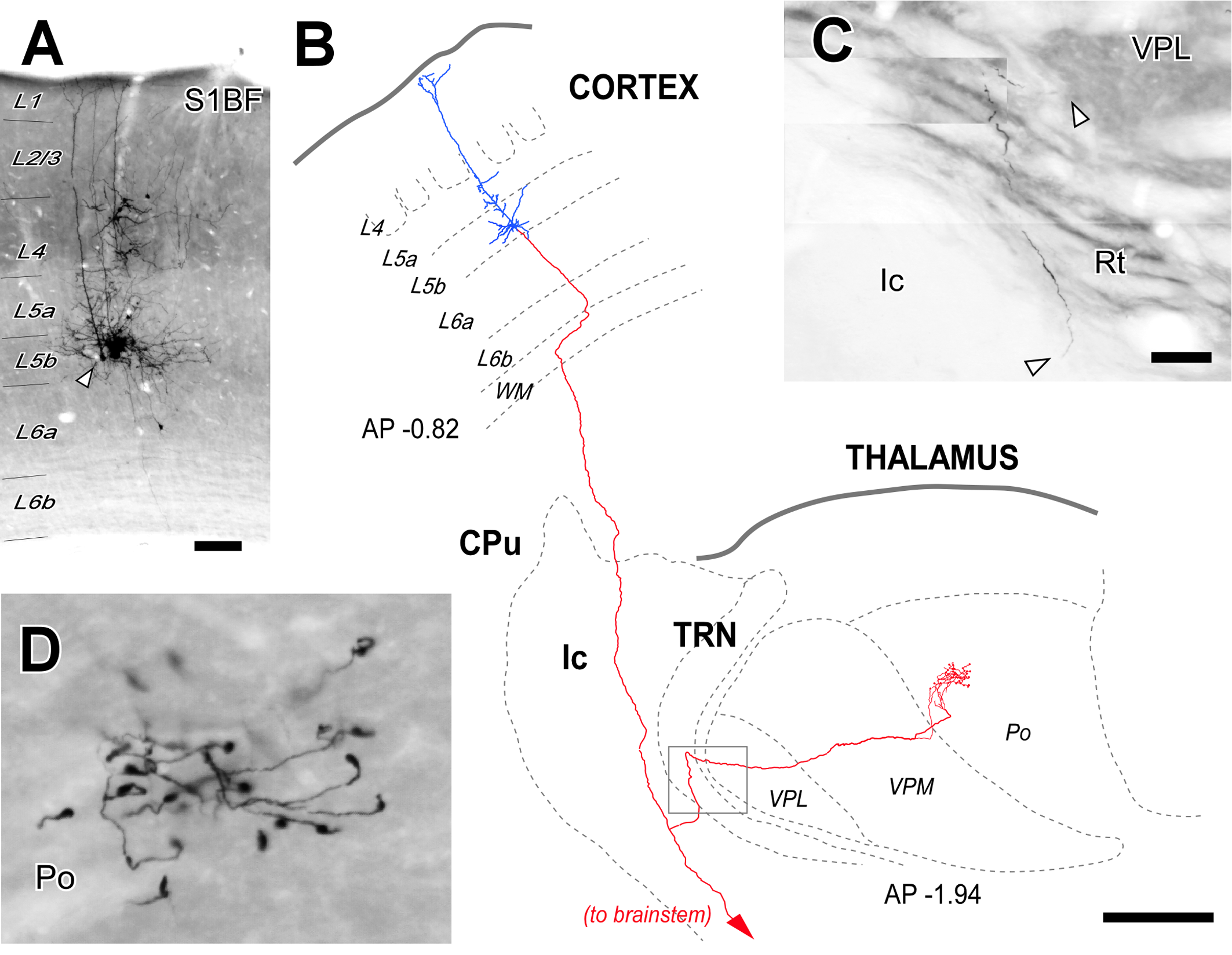

Supplement: Supplementary Data [file bhy036suppl_1.zip › Suppl_Figure6_L5singlecellreconstruction.tif]

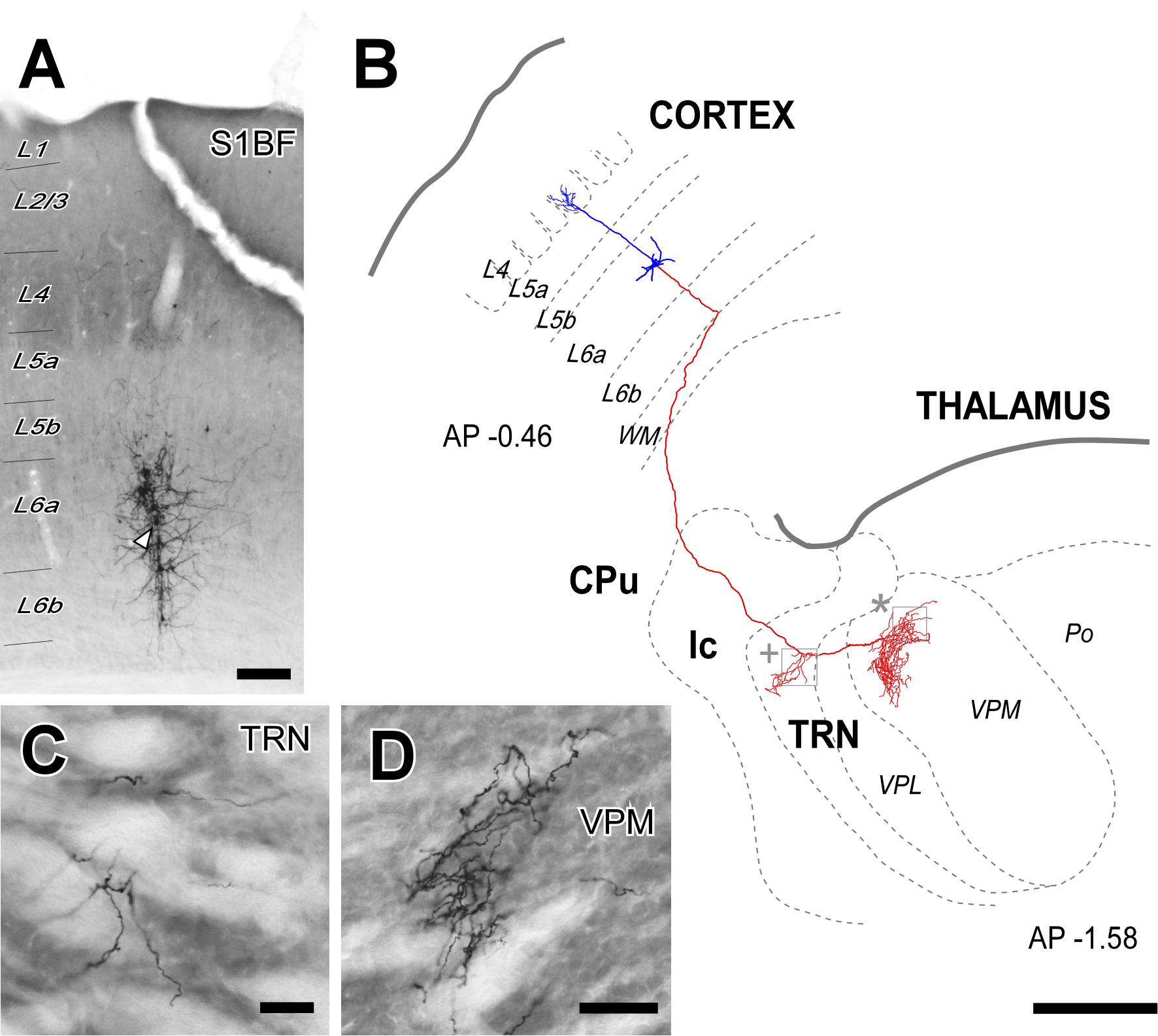

Supplement: Supplementary Data [file bhy036suppl_1.zip › Suppl_Figure7_L6asinglecellreconstruction.tif]
